# Discovery of Small Molecule Bak Activator for Lung Cancer Therapy

**DOI:** 10.7150/thno.60349

**Published:** 2021-07-25

**Authors:** Dongkyoo Park, Abu Syed Md Anisuzzaman, Andrew T. Magis, Guo Chen, Maohua Xie, Guojing Zhang, Madhusmita Behera, Gabriel L. Sica, Suresh S. Ramalingam, Taofeek K. Owonikoko, Xingming Deng

**Affiliations:** 1 Department of Radiation Oncology, Emory University School of Medicine and Winship Cancer Institute of Emory University, Atlanta, Georgia 30322, USA.; 2 Institute for Systems Biology, Seattle, WA 98109, USA.; 3 Department of Hematology and Medical Oncology, Emory University School of Medicine and Winship Cancer Institute of Emory University, Atlanta, Georgia 30322, USA.; 4 Department of Pathology and Laboratory Medicine, Emory University School of Medicine and Winship Cancer Institute of Emory University, Atlanta, Georgia 30322, USA.

**Keywords:** Bak, BH3 domain, small molecule activator, apoptosis, lung cancer, therapy

## Abstract

**Rationale:** Bak is a major proapoptotic Bcl2 family member and a required molecule for apoptotic cell death. High levels of endogenous Bak were observed in both small cell lung cancer (SCLC) and non-small cell lung cancer (NSCLC) cell lines. Increased Bak expression was correlated with poor prognosis of NSCLC patients, suggesting that Bak protein is an attractive target for lung cancer therapy. The BH3 domain functions as death domain and is required for Bak to initiate apoptotic cell death. Thus, the BH3 domain is attractive target for discovery of Bak agonist.

**Methods:** The BH3 death domain binding pocket (aa75-88) of Bak was chosen as a docking site for screening of small molecule Bak activators using the UCSF DOCK 6.1 program suite and the NCI chemical library (300,000 small molecules) database. The top 500 compounds determined to have the highest affinity for the BH3 domain were obtained from the NCI and tested for cytotoxicity for further screening. We identified a small molecule Bak activator BKA-073 as the lead compound. The binding affinity of BKA-073 with Bak protein was analyzed by isothermal titration calorimetry (ITC) assay. BKA-073-mediated Bak activation via oligomerization was analyzed by a cross-linking with Bis (maleimido) hexane (BMH). Sensitivity of BKA-073 to lung cancer cells in vitro was evaluated by dynamic BH3 profiling (DBP) and apoptotic cell death assay. The potency of BKA-073 alone or in combination with radiotherapy or Bcl2 inhibitor was evaluated in animal models.

**Results:** We found that BKA-073 binds Bak at BH3 domain with high affinity and selectivity. BKA-073/Bak binding promotes Bak oligomerization and mitochondrial priming that activates its proapoptotic function. BKA-073 potently suppresses tumor growth without significant normal tissue toxicity in small cell lung cancer (SCLC) and NSCLC xenografts, patient-derived xenografts, and genetically engineered mouse models of mutant KRAS-driven cancer. Bak accumulates in radioresistant lung cancer cells and BKA-073 reverses radioresistance. Combination of BKA-073 with Bcl-2 inhibitor venetoclax exhibits strong synergy against lung cancer in vivo.

**Conclusions:** Development of small molecule Bak activator may provide a new class of anticancer agents to treat lung cancer.

## Introduction

Lung cancer is the leading cause of cancer-related death in the United States, accounting for 24% of the total estimated cancer deaths 1, 2. Despite recent therapeutic advances, patients with advanced non-small cell lung cancer (NSCLC) eventually develop resistance to currently available cytotoxic and targeted therapy 3-5. Small cell lung cancer (SCLC) also has very limited treatment options and develops chemoresistance rapidly 6. In order to improve the survival of patients with SCLC and NSCLC, basic molecular mechanisms responsible for resistance to therapy must be carefully elucidated and such knowledge exploited for the development of more effective therapeutic agents. Apoptosis is a highly orchestrated mechanism of destroying injured and abnormal cells, which occurs in both physiological and pathological conditions 7. Impaired apoptosis is a critical step in tumor development and renders the tumor cells more resistant to conventional cytotoxic therapy 8. The decision phase of apoptosis is mainly regulated by the Bcl2 family that is composed of antiapoptotic (*i.e*. Bcl2, Bcl-XL, Mcl-1, etc.) and proapoptotic (*i.e.* Bak, Bax, Bad, Bim, Bid, PUMA, etc.) members, and mediates chemo- and radioresistance in human lung cancers 9, 10. Therefore, pharmacologic manipulation of the Bcl2 family-mediated apoptotic pathway promises to be an effective approach for cancer therapy. Indeed, the BH3-mimetic specific Bcl2 inhibitor venetoclax (ABT-199), designed to selectively bind and inhibit Bcl-2 protein, has been approved by the FDA for the treatment of patients who have relapsed or refractory chronic lymphocytic leukemia (CLL) with a genetic abnormality known as 17p deletion. However, there remains a small proportion of patients, in the range of 15-25%, who develop venetoclax failure with either venetoclax monotherapy or the venetoclax/rituximab combination 11, 12. In addition to CLL, clinical trials have demonstrated the clinical benefit of venetoclax-based therapies in newly diagnosed acute myeloid leukemia (AML), leading to the recent FDA approval of venetoclax in combination with hypomethylating agents or low-dose cytarabine for older adults with newly diagnosed AML 13. Although BH3-mimetic Bcl2 inhibitors navitoclax and venetoclax have some antitumor efficacy in SCLC preclinical models 14, 15, phase I and phase II clinical studies of single-agent navitoclax achieved limited efficacy in SCLC patients 16, 17. Therefore, it is urgent to develop new small molecules by targeting alternative Bcl2 family members for lung cancer therapy.

Bak and Bax are key multidomain proapoptotic molecules in the Bcl2 family and requisite gateways to mitochondrial dysfunction and apoptotic cell death 18. Bak is normally an integral membrane protein present on the cytosolic faces of the mitochondria and the endoplasmic reticulum, whereas Bax requires translocation from the cytosol after an apoptotic stimulus; Bak activation may thus be more tractable 19. The formation of Bak homo- or heterodimers is an important mechanism in the induction of apoptosis 20, 21. Bcl-XL and Mcl-1 can bind to Bak, which is presumably in a 'primed' conformation with its BH3 domain exposed, while in apoptosis-induced cells, a BH3-only protein displaces Bak from the anti-apoptotic heterodimer. The free Bak then forms an oligomer that elicits the permeabilization of the mitochondrial outer membrane and the release of cytochrome c (Cyt c), leading to apoptosis. Bak protein consists of nine α helices. Two central helices (α5 and α6) form the core of the protein. These two helices are predominately hydrophobic, and are flanked on one side by α3 and α4, and on the other side by α1 and α2. The BH3 binding pocket is located between α2 and α3 20, which is an ideal targeting site for the discovery of Bak agonists as potential new anti-cancer agents. Since Bak is widely expressed in both SCLC and NSCLC cells 8, 22, it provides an ideal therapeutic target for lung cancer. In this report, we have identified one lead compound Bak activator (BKA-073) that exhibits potent antitumor activity against both SCLC and NSCLC.

## Materials and Methods

### Cell lines and cell culture

All cancer cell lines used were obtained from the American Type Culture Collection (ATCC, Manassas, VA). Two months after receipt, these cell lines were employed for the described experiments without further authentication by authors. NSCLC cell lines H157, H292, H358, H460, H1299, H1972, H1975 and Calu-1 were maintained in RPMI 1640 with 5% fetal bovine serum (FBS) and 5% bovine serum (BS). NSCLC A549 cell line was cultured in F-12K medium with 10% FBS. SCLC cell lines DMS53, DMS114 and DMS153 were cultured in Weymouth's medium supplemented with 5% FBS and 5% BS as described 23. SCLC cell lines H69, H128, H146, H209 and H525 were cultured in RPMI 1640 medium supplemented with 5% FBS and 5% BS 8. The breast cancer cell lines (MDA-MB-231 and MCF7), colon cancer cell line (HCT-116) and pancreatic epithelial adenocarcinoma cell line (PANC-1) were grown in Dulbecco's modified Eagle's medium (DMEM) supplemented with 10% FBS. Lymphoma cell line (Ramos) and multiple myeloma cell lines (OPM-1 and RPMI-8226) were grown in RPMI-1640 media supplemented with 10% FBS. Sarcoma cell line U2OS was grown in McCoy's media supplemented with 10% FBS.

### Dynamic BH3 profiling (DBP)

DBP using Bim peptide was carried out as described previously 24-26. After treatment with BKA-073, venetoclax (ABT-199) or DMSO control for 16h, cells were collected, washed and resuspended at 2 x 10^6^ cells/ mL in mannitol experimental buffer (MEB) [150 mM D-mannitol, 10 mM HEPES-KOH (pH 7.5), 50 mM KCl, 20 μM EGTA, 20 μM EDTA, 0.1% BSA, and 5 mM succinate] as described 26. One volume of the 4x cell suspension was added to one volume of a 4x dye solution containing 4 μM JC-1, 40 mg/ml oligomycin, 0.02% digitonin, 20mM β-mercaptoethanol in MEB. The 2x cell/dye mixture was allowed to rest for 5-10 min at room temperature to allow for permeabilization and JC-1 staining. A total of 15μl of the 2x cell/dye mixture was loaded into a 384-well plate that contained 15µL of Bim BH3 peptide (0.03, 0.1, 0.3, 1, and 3 μM) in MEB, shaken for 15 s inside the reader, and the fluorescence at 590 nm monitored every 5 min for 180 min at 32 °C. The dynamic BH3 profile (∆ % Priming) was calculated as described 24-26.

### Isothermal titration calorimetry (ITC)

The binding affinity of BKA-073 with Bak protein was examined by isothermal titration calorimetry (ITC) assay as described 27, 28. ITC assay was carried out in the auto-iTC200 instrument (MicroCal, GE) at 25^o^C. Purified recombinant human or mouse Bak protein was loaded into a 96 Deepwell PP plate. BKA-073 compound was then titrated stepwise into the protein for a total of 16 injections. Reference power and initial delay were set as 5 µCal/sec and 60s, respectively. A string speed of 750 rpm was used for the ITC measurements. The binding constant (K_d_) value was determined by fitting the titration curve to a one-site binding mode, using the Origin software provided by the manufacturer.

### Bak oligomerization

Bak oligomerization was analyzed as described 19. Briefly, 10 mM Bis (maleimido) hexane (BMH) was added to the mitochondrial fraction dissolved in conjugation buffer (PBS, pH7.2) and 5mM EDTA was added for crosslinking between sulfhydryl groups of Bak proteins. The reaction mixture was incubated for 1h at room temperature. The reaction was stopped by adding quench solution (1M DTT) for 15min at room temperature. The reaction product was subjected to SDS-PAGE gel and analyzed by Western blot using a Bak antibody.

### Patient samples

Use of human lung cancer tumor tissues for IHC was approved by the Institutional Review Board of Emory University (IRB approval number 20081986). Paraffin-embedded human lung tissue samples from 208 NSCLC patients were obtained from the tissue bank at Emory University Winship Cancer Institute. Tissue microarray (TMA) was constructed with replicate cores of tumor and adjacent normal lung.

### Lung cancer xenografts and treatments

Six-week-old female Nu/Nu nude mice were purchased from Envigo (Indianapolis, IN) and housed under pathogen-free conditions in microisolator cages. The animal propagation protocol was approved by the Emory Institutional Animal Care and Use Committee (IACUC) and the Emory Animal Ethics Committee. 3×10^6^ lung cancer cells were injected into subcutaneous tissue in the flank region of nude mice. The tumors were allowed to grow to an average volume of 150~200 mm^3^ prior to initiation of therapy as previously described 28, 29. Patient-derived xenografts (PDXs, TKO-002 and TKO-005) were established as part of an IRB-approved phase II co-clinical trial of arsenic trioxide in patients with relapsed SCLC who had failed standard platinum-based chemotherapy. Tumor samples were obtained by image-guided biopsy and were directly implanted into Nu/Nu nude mice without intervening propagation in plastic culture plates. Direct serial propagation of growing tumor occurred for up to 5 generations. The animal propagation protocol was approved by the Emory IACUC and the Emory Animal Ethics Committee. Mice with SCLC and NSCLC xenografts or PDXs were treated with BKA-073 intraperitoneally (i.p.) or venetoclax orally as indicated. During treatment, tumor volume (V) was measured by caliper measurements once every two days as described previously 29. Mice were euthanized by CO_2_ inhalation at the end of treatment. Harvested tumor tissues were used for further analysis.

### Genetically engineered mouse model (GEMM), treatment and tumor burden quantification

LSL-KRAS^G12D^ LKB1^fl/fl^ (KL) were generated as previously described 30-33. All studies were performed on protocols approved by the Emory University IACUC. Six weeks after cre-adenovirus infection, KL mice were treated with BKA-073 intraperitoneally (i.p) for 48 days. Mice were euthanized with CO_2_ asphyxiation. After lung perfusion with PBS, the left lung lobes were harvested from mice in control and BKA-073 treated groups and immediately fixed in 10% neutral buffered formalin (Fisher Scientific, Kalamazoo, MI), horizontally cut into three equal parts and embedded in paraffin blocks. Three parts of lung tissues representing different regions of the lung were vertically put into paraffin blocks for hematoxylin-eosin (H&E) staining. Lung tissue samples were sectioned at 3 µm three times for placement of slides and stained with H&E. H&E stained samples were scanned using Nano Zoomer 2.0-HT (Hamamatsu, Japan) and images were analyzed using ImageScope viewing software (Leica Biosystems, Buffalo Grove, IL). Tumor numbers were counted under a microscope and tumor area was quantified using Openlab modular imaging software (PerkinElmer, Waltham, MA) as previously described 31, 32.

### Statistical analysis

All data are presented as mean ± standard deviation (s.d.) from at least three independent experiments. The statistical significance of differences between groups was analyzed by two-tailed t test. We chose the sample size to detect a minimum effect size of 1.5 with at least 80% power and a type I error of 0.05 for each comparison. The log rank test was used to test differences in the Kaplan-Meier survival assay. A value of *P* < 0.05 was considered to be statistically significant.

## Results

### Screening of small molecules targeting the BH3 binding pocket of Bak for lung cancer therapy

**Figure 1:**
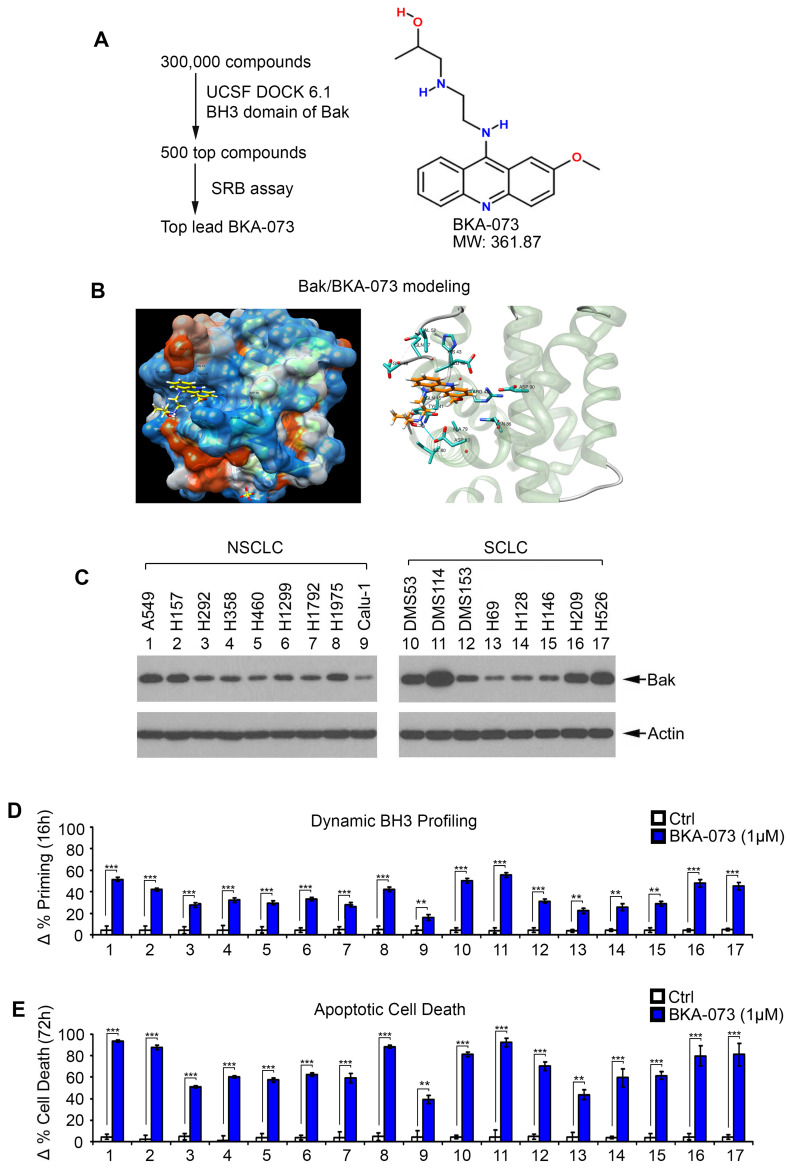
** Discovery of small molecule BKA-073 as a lead compound that targets BH3 domain of Bak and induces mitochondrial priming and apoptosis in lung cancer cells.** (**A**) Schematic illustration of screening strategies used to identify the lead Bak agonist BKA-073 and its chemical structure. (**B**) Structural modeling of BKA-073 in the BH3 domain binding pocket of Bak protein. (**C**) Expression levels of Bak were analyzed by Western blot in NSCLC and SCLC cell lines. (**D-E**) A panel of NSCLC and SCLC cell lines were treated with BKA-073 (1μM) for 16h or 72h, followed by analysis of dynamic BH3 profiling (D) or apoptotic cell death (E), respectively. Data represent the mean ± SD, *n* = 3 per group. **P < 0.01, ***P < 0.001, by 2-tailed *t* test.

The BH3 death domain is known to be required for the proapoptotic function of Bak 19. We chose the BH3 domain binding pocket (aa75-88) of Bak (PDB ID: 2YV6) as a docking site for screening of small molecules using the UCSF DOCK 6.1 program suite and the NCI chemical library (300,000 small molecule) database as we recently described 8, 20, 29, 34. Small molecules were ranked according to their energy scores. The top 500 compounds determined to have the highest affinity for the BH3 domain were obtained from the NCI and tested for cytotoxicity in human lung cancer cells (*i.e*. H1299, H460 and A549 cells) by sulforhodamine B (SRB) assay for further screening as described 35, 36. Among these small molecules, one compound (NSC14073) had the most potent activity against human lung cancer cells. We named this lead compound Bak activator BKA-073 (C_19_H_24_ClN_3_O_2_, MW: 361.87) (**Figure 1A**). The molecular modeling of this lead in complex with Bak is shown in **Figure 1B**. To test the effect of BKA-073 on mitochondrial priming (∆% priming) and apoptotic cell death, human lung cancer A549 cells were treated with increasing concentrations (0, 0.25, 0.5, 0.75, 1.0 µM) of BKA-073, followed by analysis of dynamic BH3 profiling (DBP) at 16h and apoptotic cell death at 72h as described 24, 37. DBP is a functional assay that can measure early changes in net pro-apoptotic signaling at the mitochondrion (''priming'') induced by chemotherapeutic agents or targeted agents in cancer cells 25. Priming is a measure of how close a cell is to the threshold of apoptosis. Results indicated that BKA-073 induced mitochondrial priming and apoptosis in a dose-dependent manner (**Figure S1**). To extend this finding, a panel of NSCLC and SCLC cell lines was tested. BKA-073 potently induced mitochondrial priming and apoptosis in both NSCLC and SCLC cell lines that express various levels of endogenous Bak (**Figure 1C, D** and** E**). Intriguingly, NSCLC cell lines (*i.e.* A549, H157 and H1975) and SCLC cell lines (*i.e*. DMS53, DMS114, H209 and H526) that express relatively higher levels of Bak were more sensitive to BKA-073. In contrast, lung cancer cell lines expressing relatively lower levels of endogenous Bak (*i.e*. NSCLC cell line: Calu-1; SCLC cell lines: H69, H128 and H146) were less sensitive to BKA-073 (**Figure 1C, D** and** E**). Thus, the sensitivity of BKA-073 induction of mitochondrial priming and apoptosis is relatively dependent on Bak expression level. In addition to lung cancer cell lines, we evaluated the efficacy of BKA-073 in other types of cancer cell lines, including breast cancer (MDA-MB-231 and MCF7), colon cancer (HCT-116), lymphoma (Ramos), multiple myeloma (OPM-1), pancreatic cancer (PANC-1) and osteosarcoma (U2OS) cell lines. Results revealed that BKA-073 also potently induced mitochondrial priming and apoptotic cell death in various types of cancer cell lines (**Figure S2**), suggesting that BKA-073 should be effective in various cancer types.

### BKA-073 directly binds to Bak protein and induces Bak oligomerization leading to Cyt c release

**Figure 2:**
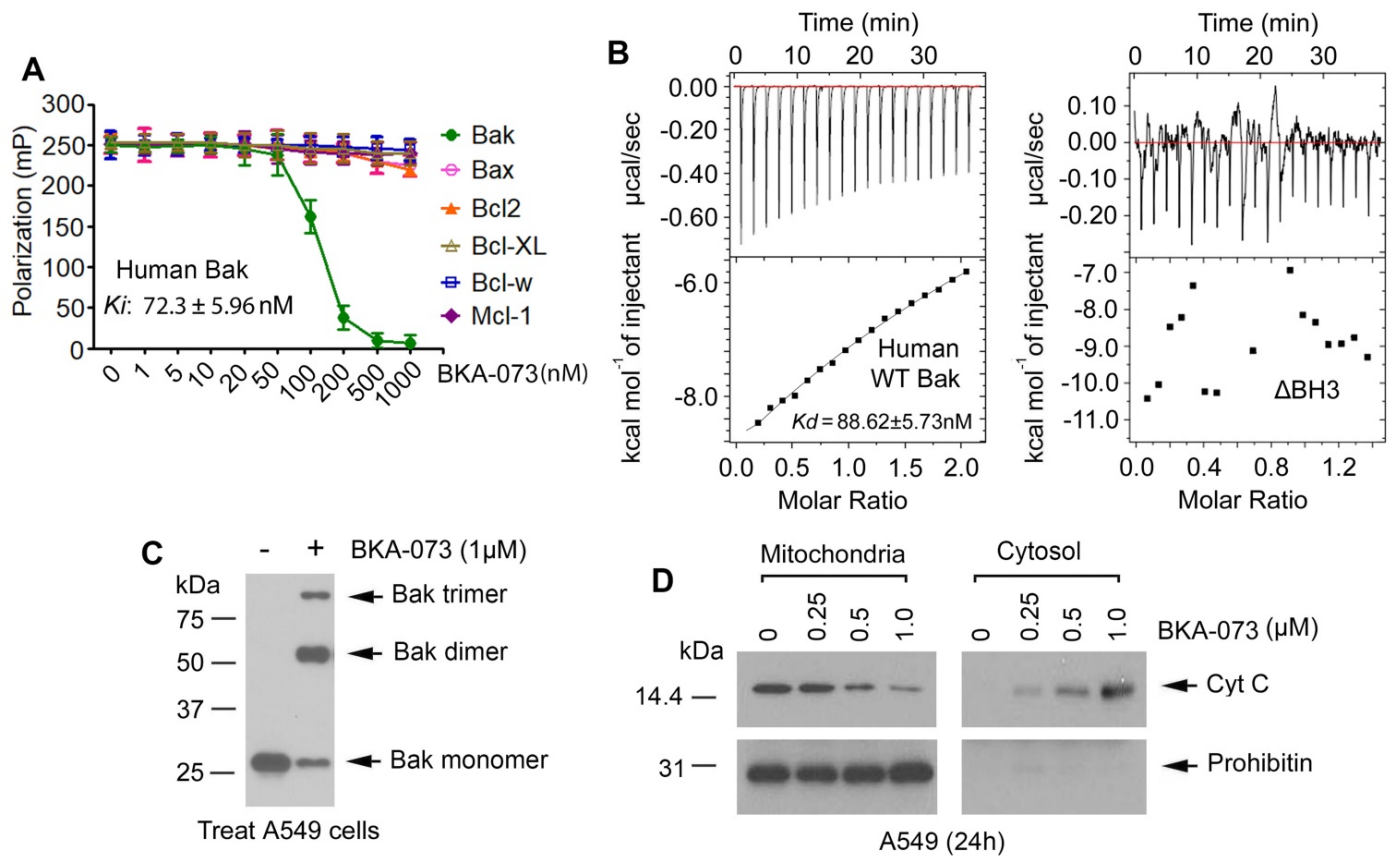
** BKA-073 specifically binds to Bak, induces Bak oligomerization and Cyt c release in lung cancer cells.** (**A**) Fluorescence polarization assay was performed to measure the inhibitory constant (*K*i) value using purified Bak protein or other Bcl2 family member(s), BKA-073, and fluorescence-labeled Bak BH3 peptide. Data represent the mean ± SD, *n* = 3 per group. (**B**) The binding affinity of BKA-073 with WT Bak or ∆BH3 Bak deletion mutant protein was examined by isothermal titration calorimetry assay. The binding constant (*K*_D_) value was determined by fitting of the titration curve to a 1-site binding mode. (**C**) A549 cells were treated with BKA-073 (1μM) for 24h, followed by mitochondrial isolation and crosslinking using BMH. Bak was analyzed by western blot. (**D**) A549 cells were treated with increasing concentrations of BKA-073 for 24h. Mitochondrial and cytosolic fractions were isolated. Levels of Cyt c in these two fractions were analyzed by Western blot. Prohibitin was used as a mitochondrial marker for purity of the subcellular fractions.

To confirm the binding of BKA-073 with Bak, we first conducted a competitive fluorescence polarization (FP) assay using purified human Bak protein, fluorescent Bak BH3 domain peptide, and BKA-073 as described 8, 37-40. BKA-073 directly bound to human Bak protein with high binding affinity (*Ki*: 72.3 ± 5.96 nM). Specifically, BKA-073 had very low binding affinity with other Bcl2 family members (**Figure 2A**), indicating that it selectively binds to Bak. There are multiple amino acid differences in the BH3 domains between Bak and other Bcl2 family members 20, which helps to explain why BKA-073 only binds to Bak but not to other Bcl2 family members (*i.e*. Bax, Bcl2, Bcl-XL, Bcl-w and Mcl-1). In addition to the FP assay, isothermal titration calorimetry (ITC) was also employed to measure Bak/BKA-073 binding. ITC is a direct, label- and immobilization-free technique which measures the binding affinity between proteins and small molecule ligands that interact with each other, and can analyze binding constant (K_d_) values in the millimolar and nanomolar range 41, 42. We performed ITC experiments to assess BKA-073/Bak binding using an auto-iTC200 instrument as described 27, 43. Results revealed that BKA-073 directly bound human Bak protein with nanomolar range binding affinity (K_d_ = 88.62 ± 5.73 nM) (**Figure 2B,** left panel). In contrast, BKA-073 failed to bind to the BH3 deletion human Bak mutant protein (ΔBH3) in the ITC assay (**Figure 2B**, right panel), suggesting that the BH3 domain is essential for Bak to interact with BKA-073. In addition to human Bak/BKA-073 binding, we measured mouse Bak/BKA-073 binding using ITC. Intriguingly, BKA-073 also directly bound mouse Bak protein with good binding affinity (K_d_ = 93.37 ± 7.91 nM) (**Figure S3**). Our findings suggest that BKA-073 can bind both human and mouse Bak proteins.

A pivotal step in the apoptosis process is the oligomerization of Bak 19, 44. To assess whether BKA-073 affects the ability of Bak to form oligomers in the mitochondrial membrane, a cross-linking study with Bis (maleimido) hexane (BMH) was carried out. Intriguingly, treatment of A549 cells with BKA-073 (1μM) facilitated the formation of Bak dimers and trimers (**Figure 2C**). The molecular sizes of these adducts were estimated to be multiples of ∼28 kDa, suggesting the formation of Bak homo-oligomers in A549 cells. These findings indicate that BKA-073 can activate Bak through its oligomerization in mitochondria. It is known that formation of Bak oligomers in mitochondria promotes cytochrome c (Cyt c) release to induce apoptosis 45, 46. Intriguingly, BKA-073-induced Bak oligomerization promoted Cyt c release from mitochondria in A549 cells (**Figure 2D**).

### Bak is a required target for BKA-073 induction of mitochondrial priming, apoptosis and Cyt c release

**Figure 3:**
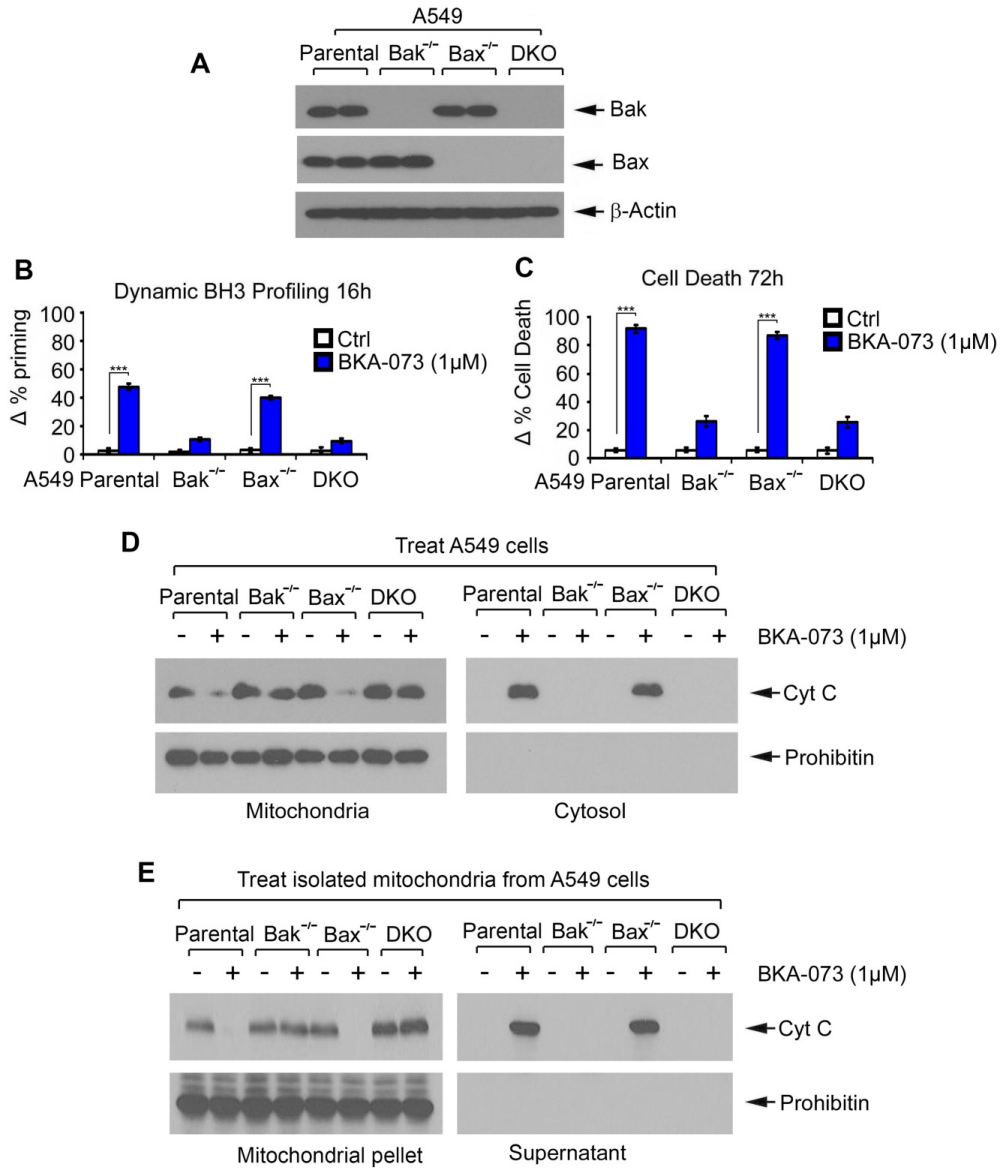
** Bak is required for BKA-073 induction of mitochondrial priming, Cyt c release and apoptotic cell death.** (**A**) Levels of Bak and Bax were analyzed by Western blot in parental, Bak ^-/-^, Bax ^-/-^ or Bax/Bak double-knockout (DKO) A549 cells. (**B-C**) Parental, Bak ^-/-^, Bax ^-/-^ or DKO A549 cells were treated with BKA-073 (1μM) for 16h or 72h, followed by analysis of dynamic BH3 profiling (B) or apoptotic cell death (C), respectively. Data represent the mean ± SD, *n* = 3 per group. ***P < 0.001, by 2-tailed *t* test. (**D**) Parental, Bak ^-/-^, Bax ^-/-^ or DKO A549 cells were treated with BKA-073 (1μM) for 24 h. Mitochondrial and cytosolic fractions were isolated. Levels of Cyt c in these two fractions were analyzed by western blot. (**E**) Mitochondria were isolated from parental, Bak ^-/-^, Bax ^-/-^ or DKO A549 cells. The isolated mitochondria were then treated with BKA-073 (1μM) in mitochondrial buffer for 30 min at 30^o^C. After centrifugation, Cyt c in supernatant fraction (i.e. Cyt c release) and Cyt c in mitochondrial pellet were analyzed by western blot.

To further test whether Bak or Bax is an essential target for BKA-073 induction of mitochondrial priming and apoptotic cell death, first, A549 Bak^-/-^, A549 Bax^-/-^ and A549 Bak^-/-^ Bax^-/-^ (double knockout, DKO) cells were generated using CRISPR/Cas9 as described in “Methods”. Knockout of Bak, Bax or both was confirmed by Western blot (**Figure 3A**). Parental, Bak^-/-^, Bax^-/-^ and DKO A549 cells were then treated with BKA-073 (1μM) for 16h or 72h, followed by analysis of DBP or apoptotic cell death, respectively. Bak^-/-^ and DKO A549 cells exhibited significant resistance to BKA-073 while parental and Bax^-/-^ A549 cells were sensitive to BKA-073 (**Figure 3B** and** C**), indicating that Bak, but not Bax, is a required target through which BKA-073 induces apoptosis.

To further test whether Bak is required for BKA-073 induction of Cyt c release, A549 parental, Bak^-/-^, Bax^-/-^ and DKO cells were treated with BKA-073 (1 μM) for 24h, followed by analysis of Cyt c release from mitochondria. Results revealed that Cyt c release was observed in A549 parental and Bax^-/-^ cells but not in A549 Bak^-/-^ and DKO cells (**Figure 3D**). To further test whether BKA-073 directly induces Cyt c release from isolated mitochondria in a Bak- but not Bax-dependent fashion, purified mitochondria were isolated from A549 parental, Bak^-/-^, Bax^-/-^ and DKO cells. The isolated mitochondria were treated with BKA-073 (1 μM) for 30 min at 30 ^o^C. After centrifugation at 13000 g for 5 min, Cyt c in the supernatant (i.e., Cyt c release) was analyzed by Western blot. Results showed that BKA-073 induced Cyt c release from the mitochondria isolated from A549 parental and Bax^-/-^ cells, but not from Bak^-/-^ or DKO A549 cells, indicating that BKA-073 induces Cyt c release from isolated mitochondria in a Bak- but not Bax-dependent manner (**Figure 3E**).

### The BH3 domain is required for BKA-073 induction of mitochondrial priming and apoptotic cell death

The apoptotic effect of BKA-073 is dependent on Bak expression since A549 Bak knockout cells are resistant to BKA-073 (**Figure 3**). Importantly, BKA-073 can bind WT but not the BH3 deletion mutant (ΔBH3) Bak protein in ITC assay (**Figure 2B**), suggesting that the BH3 domain is essential for Bak to interact with BKA-073. To further test whether BKA-073/Bak binding is required for BKA-073 induction of mitochondrial priming and apoptosis, WT and ΔBH3 mutant Bak were exogenously overexpressed in A549 Bak^-/-^ cells (**Figure S4A**). Cells were then treated with BKA-073 for 16h and 72h, followed by analysis of dynamic BH3 profiling and apoptotic cell death, respectively. Results indicated that A549 Bak^-/-^ cells transfected with vector-only were resistant to BKA-073. Exogenous expression of WT Bak restored sensitivity of cells to BKA-073 (**Figure S4B, C**). However, exogenous expression of the ΔBH3 mutant Bak in A549 Bak^-/-^ cells failed to restore sensitivity to BKA-073 (**Figure S4B, C**). These results indicate that the BH3 domain is critical for BKA-073 induction of mitochondrial priming and apoptotic cell death in human lung cancer cells. Structural modeling analysis by computational programming revealed that BKA-073 interacts with amino acids Ala79, Ile80, Asp83 and Asn86 in the BH3 domain of Bak protein. We generated a panel of Bak mutants within the BH3 domain at the specific residues identified by the initial docking simulations, including A79E, I80E, D83E and N86E Bak mutants. To test which BKA-073 binding amino acid(s) in the BH3 domain are critical for BKA-073 induction of apoptosis, WT and all Bak mutants were exogenously overexpressed in A549 Bak^-/-^ cells (**Figure S5A**). Cells were then treated with BKA-073 for 16h and 72h, followed by analysis of dynamic BH3 profiling and apoptosis, respectively. Results indicated that exogenous expression of WT Bak, A79E or I80E in A549 Bak^-/-^ cells completely or partially restored sensitivity of cells to BKA-073 (**Figure S5B, C**). However, exogenous expression of D83E or N86E in A549 Bak^-/-^ cells failed to restore sensitivity to BKA-073 (**Figure S5B, C**). These results indicate that D83 and N86 sites in the BH3 domain are critical for BKA-073 induction of apoptosis in human lung cancer cells.

### BKA-073 potently suppresses NSCLC xenografts via induction of apoptosis in a Bak-dependent manner

**Figure 4:**
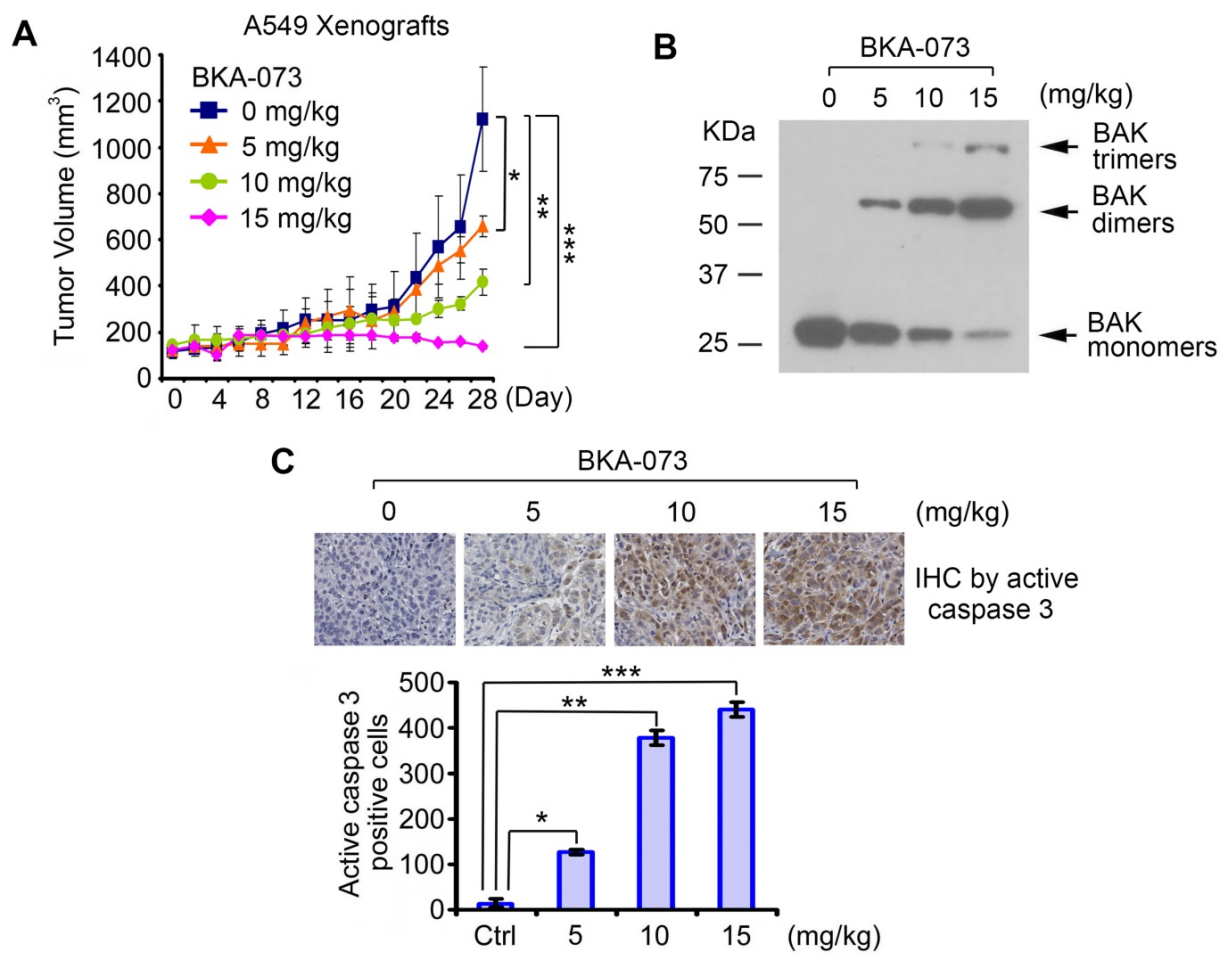
** BAK-073 potently suppresses lung cancer growth in a dose-dependent manner *in vivo****.* (**A**) Nu/Nu mice bearing A549 lung cancer xenografts were treated with increasing doses of BKA-073 (5~15mg/kg/d) i.p. for 28 days. Tumor volume was measured once every 2 days. After treatment, mice were sacrificed and tumors were removed and analyzed. Data represent the mean ± SD, *n* = 8 per group. *P < 0.05, **P < 0.01, ***P < 0.001, by 2-tailed *t* test. (**B**) After treatment of mice with BKA-073, mitochondria were isolated from tumor tissues, followed by cross-linking with BMH. Bak oligomerization was analyzed by Western blot. (**C**) Active caspase 3 was analyzed in tumor tissues at the end of experiments by IHC staining. Data represent the mean ± SD, *n* = 8 per group. *P < 0.05, **P < 0.01, ***P < 0.001, by 2-tailed *t* test.

**Figure 5:**
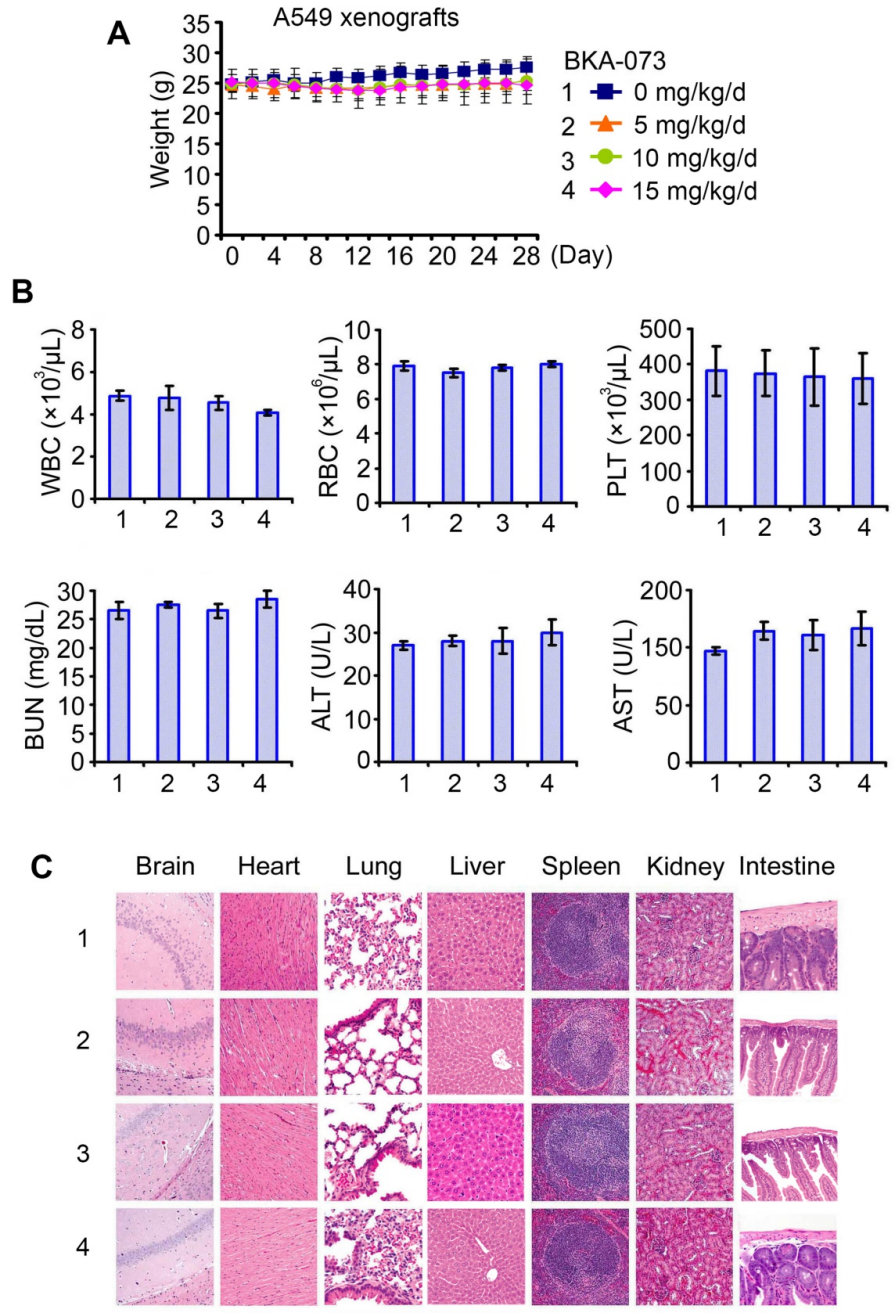
** Toxicity of BKA-073 *in vivo****.* (**A-C**) Body weight (A), blood analysis (B) and H&E histology of various organs (C) from mice bearing A549 xenografts after treatment with increasing doses (0, 5, 10, 15mg/kg/d) of BKA-073 for 28 days.

To test the potency of BKA-073 *in vivo*, mice carrying lung cancer xenografts derived from A549 cells were treated i.p. with increasing doses (0, 5, 10, 15mg/kg/d) of BKA-073 for 28 days. BKA-073 potently suppressed lung cancer growth in a dose-dependent fashion (**Figure 4A**). To assess whether BKA-073 induced suppression of tumor growth occurs through activation of Bak and apoptosis *in vivo*, representative samples from harvested tumor tissues were analyzed by cross-linking with BMH for Bak oligomerization or by immunohistochemistry (IHC) for active caspase 3 as described 19, 29, 47. Dose-dependent Bak oligomerization and apoptosis were observed in tumor tissues after BKA-073 treatment (**Figure 4B, C**). Importantly, doses of 5-15mg/kg/d not only potently suppressed tumor growth but were also well tolerated without significant toxicity to mice. No weight loss was observed in each treatment group (**Figure 5A**). There were no decreases in white cells (WBC), red blood cells (RBC) and platelets (PLT) in blood. Tests of kidney (BUN) and liver (ALT and AST) function were in the normal range (**Figure 5B**). Histopathology of harvested normal tissues (heart, liver, lung, brain, spleen, kidney, intestine, etc.) revealed no evidence of normal tissue toxicities after treatment with doses of 5~15mg/kg/d (**Figure 5C**). These findings suggest that doses between 5mg/kg and 15mg/kg provide the optimal therapeutic index for BKA-073 for *in vivo* experimentation involving lung cancer xenografts.

**Figure 6:**
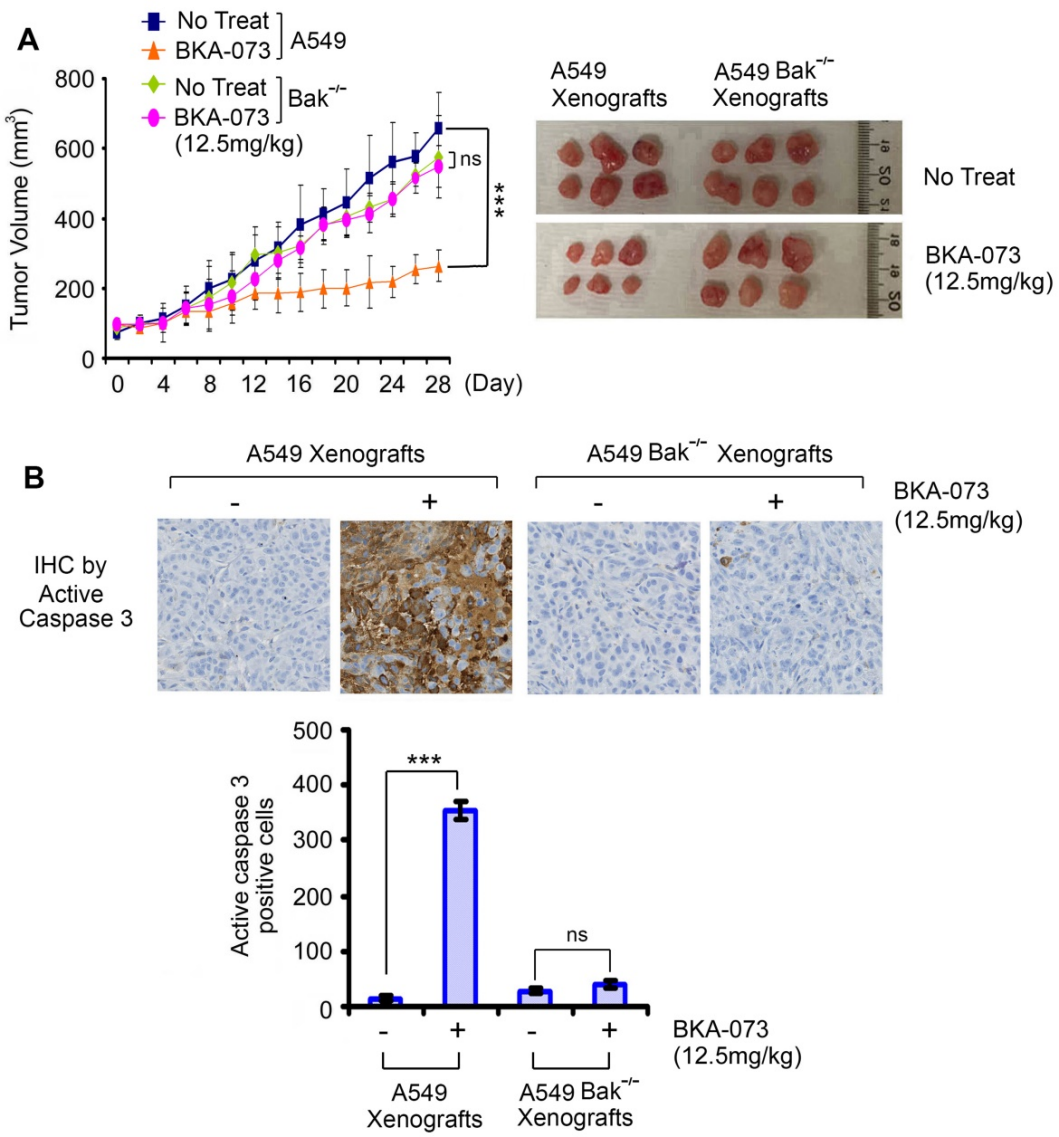
** Bak expression is required for BKA-073 suppression of lung cancer growth *in vivo***. (**A**) Nu/Nu mice carrying parental A549 xenografts or Bak^-/-^ A549 xenografts were treated with BKA-073 (12.5mg/kg/d) i.p. for 28 days. Tumor volume was measured once every 2 days. After treatment, mice were sacrificed and tumors were removed and analyzed. Data represent the mean ± SD, *n* = 6 per group. ***P < 0.001, ns: not significant, by 2-tailed *t* test. (**B**) Active caspase 3 was analyzed in tumor tissues at the end of experiments by IHC staining. Data represent the mean ± SD, *n* = 6 per group. ***P < 0.001, ns: not significant, by 2-tailed *t* test.

To test whether Bak expression is required for BKA-073 suppression of lung cancer growth *in vivo*, mice carrying xenografts derived from A549 parental or Bak^-/-^ cells were treated i.p. with BKA-073 (12.5mg/kg/d) for 28 days. Intriguingly, BKA-073 potently inhibited growth of A549 xenografts but had no inhibitory effect on Bak^-/-^ A549 xenografts (**Figure 6**), indicating that BKA-073 suppression of NSCLC xenografts occurs in a Bak-dependent manner.

### BKA-073 exhibits potent antitumor activity against SCLC in xenografts and PDX models

**Figure 7:**
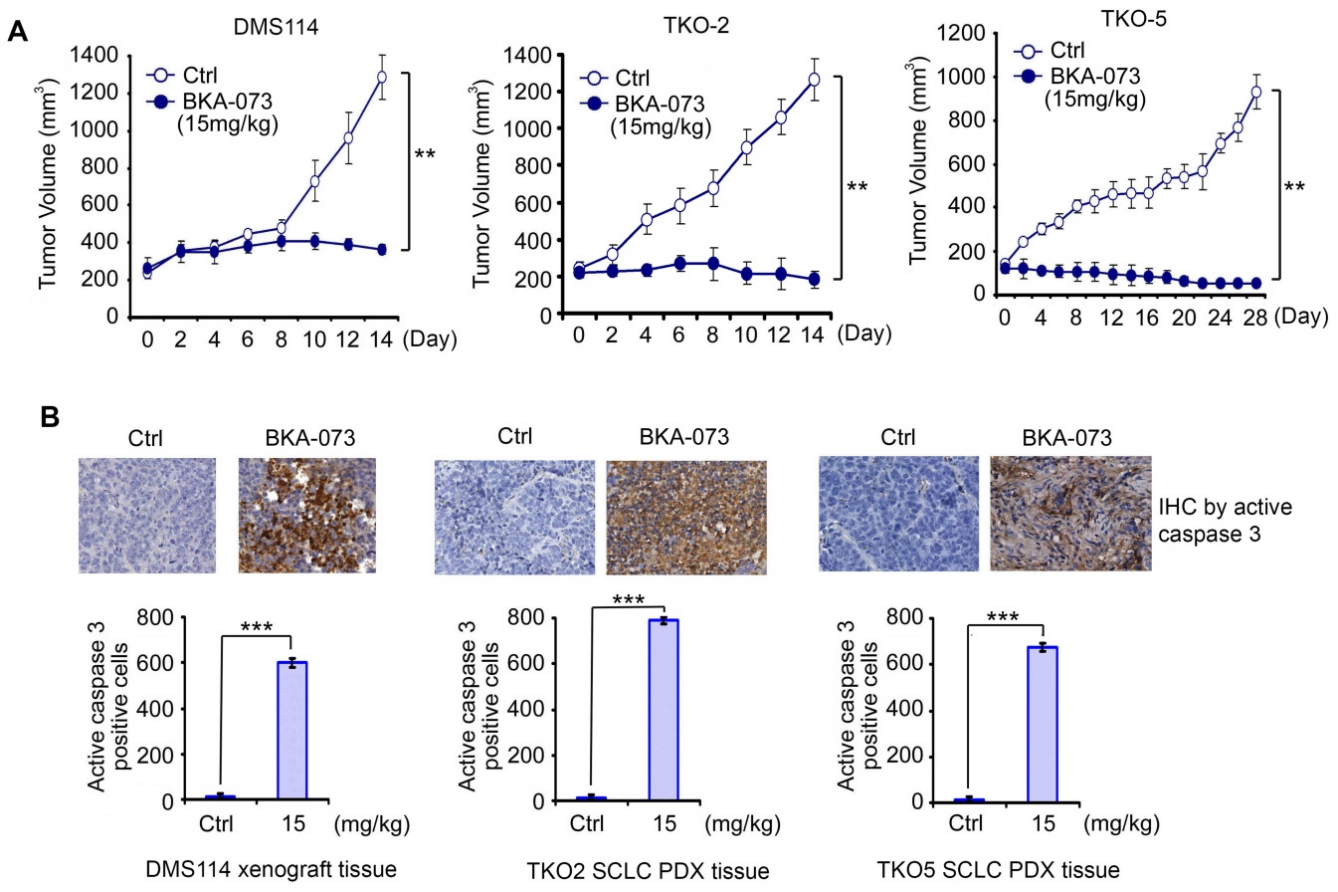
** BKA-073 suppresses SCLC in xenografts and PDX models**. (**A**) Nu/Nu mice bearing xenografts derived from SCLC cell line DMS114 or from SCLC patients (TKO-2 or TKO-5) were treated with BKA-073 (15mg/kg/d) i.p. for 14 or 28 days. Tumor volume was measured once every 2 days. After treatment, mice were sacrificed and tumors were removed and analyzed. Data represent the mean ± SD, *n* = 8 per group. **P < 0.01, by 2-tailed *t* test. (**B**) Active caspase 3 was analyzed in tumor tissues at the end of experiments by IHC staining. Data represent the mean ± SD, *n* = 8 per group. ***P < 0.001, by 2-tailed *t* test.

In addition to NSCLC cell lines, BKA-073 also efficiently suppressed the growth of SCLC cell lines that express high levels of endogenous Bak (**Figure 1**). To further evaluate the anti-tumor activity of BKA-073 against SCLC *in vivo*, mice carrying SCLC xenografts derived from the DMS114 cell line or patient-derived xenografts (PDXs) from two patients with refractory SCLC (TKO-2 and TKO-5) 48 were treated i.p. with BKA-073 (15mg/kg/d) for 2-4 weeks. BKA-073 potently suppressed tumor growth of DMS114 xenografts and SCLC PDXs, which occurred through induction of apoptosis (**Figure 7**). PDX models are expected to better recapitulate the SCLC tumor setting without intervening *in vitro* culture. These findings indicate that BKA-073 may potentially be effective in patients with SCLC, for which there are currently limited treatment options.

### BKA-073 represses mutant KRAS-driven lung cancer growth and prolongs survival in genetically engineered mouse models (GEMMs)

**Figure 8:**
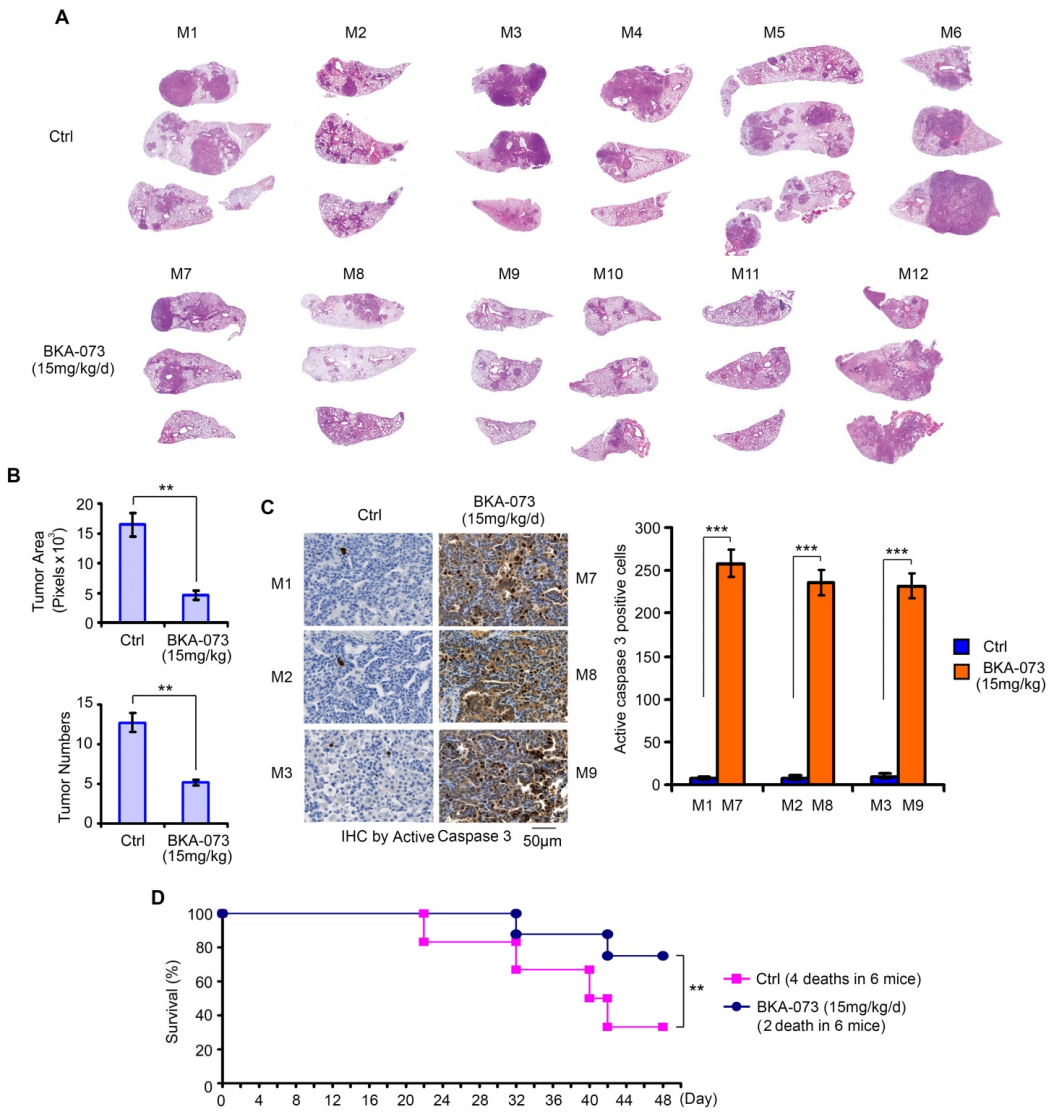
** BKA-073 suppresses lung cancer growth and prolongs survival in GEMMs**. (**A**) After administration of adenovirus Cre recombinase in KRASG12D LKB1fl/fl (KL) mice for 6 weeks, KL mice were treated with KRA-073 (15mg/kg/d) i.p. for 48 days (n=6 mice/group). H&E images from control or treatment group are shown. (**B**) Tumor numbers were counted under the microscope and tumor area was quantified using Openlab modular imaging software. Data represent mean ± SD, n = 6 per group. **P < 0.01, by 2-tailed t test. (**C**) Active caspase 3 was analyzed in tumor tissues from three representative control mice and three treated mice at the end of experiments by IHC staining. Data represent the mean ± SD, *n* = 3 per group. ***P < 0.001, by 2-tailed *t* test. (**D**) Survival of mice was calculated up to 48 days before euthanization in the control group versus the BKA-073 treatment group. Data represent mean ± SD, n = 6 per group, **P < 0.01, by 2-tailed t test.

KRAS is the most commonly mutated oncogene, yet no effective targeted therapies exist for KRAS-mutant cancers 49. Intriguingly, expression of exogenous constitutively active KRAS (G12D) mutant in H1944 cells with wild-type KRAS background significantly enhanced Bak expression (**Figure S6A**). Since BKA-073 is able to induce apoptosis by activation of Bak via facilitating its oligomerization *in vitro* and *in vivo* (**Figures 1, 2 and 4**), we were interested in testing whether BKA-073 is effective for the treatment of mutant KRAS-driven cancer. To assess the potency of BKA-073 in KRAS mutant-driven lung cancer, lox-stop-lox (LSL*)*-KRAS^G12D^ LKB1^fl/fl^ (*i.e*. KL) mice were generated and bred out as previously described 30, 31, 50, 51. These mice contain a KRAS^G12D^ LSL knock-in allele and a floxed allele of LKB1 (LKB1^fl/fl^) 30, 31. Primary lung adenocarcinoma was detectable as early as 6 weeks after intranasal administration of 5×10^6^ pfu adenovirus expressing Cre recombinase (AdeCre) in KRAS^G12D^ LKB1^fl/fl^ (KL) mice. Intriguingly, increased Bak expression was observed in tumor tissues from KL mice as compared to adjacent normal lung tissues (**Figure S6B**), thereby providing a strong rationale to employ Bak agonist BKA-073 for the treatment of mutant KRAS-driven lung cancer. To further assess the potential of BKA-073 as therapy for mutant KRAS-driven lung cancer, BKA-073 (15 mg/kg/d) or vehicle was administered to KL mice i.p. starting at 6 weeks post AdeCre delivery as previously suggested 31-33. After treatment for 48 days, KL mice were euthanized with CO_2_ asphyxiation. Lungs with tumor and normal lung tissues were collected for further analysis. To quantify tumor burden and tumor multiplicity in mice, H&E-stained lungs were imaged with morphometric software to quantify the surface area composed of tumor as opposed to normal tissue of representative cross-sections of each lung lobe for each mouse as previously described 31-33. Results indicated that treatment of KL mice with BKA-073 resulted in significant reduction of tumor burden and multiplicity in the lung via apoptosis (**Figure 8A, B** and** C**). Importantly, treatment with BKA-073 significantly prolonged survival of KL mice when compared with the control group (**Figure 8D**). There were 4 deaths out of 6 mice in the control group versus 2 death out of 6 mice in the BKA-073 treatment group (p < 0.01), calculated up to 48 days before euthanization.

### Bak accumulates in radioresistant lung cancer cells and BKA-073 reverses radioresistance in vitro and in vivo

To further investigate whether Bak contributes to radioresistance, we established three lung cancer cell lines with ionizing radiation resistance (*i.e*. A549-IRR, H358-IRR and H460-IRR) as described 8, 52, 53. Increased levels of Bak were observed in A549-IRR, H358-IRR and H460-IRR cells as compared to parental A549 (A549-P), H358 (H358-P) and H460 (H460-P) cells (**Figure S7A**). A549-IRR, H358-IRR and H460-IRR cells grow well under cell culture conditions, indicating that Bak molecules are in an inactive form under normal growth conditions. A549-P, H385-P and H460-P cells remained sensitive to IR, but A549-IRR, H358-IRR and H460-IRR became insensitive to IR. Intriguingly, both parental and radioresistant cell lines were sensitive to BKA-073 (**Figure S7B**), suggesting that BKA-073 is efficacious in radioresistant cells. To further test this *in vivo*, NSCLC xenografts derived from A549-P and A549-IRR cell lines were treated with IR (2Gy/exposure, every other day for total of 5 times) or BKA-073 (15 mg/kg/d) for 4 weeks. We observed that lung cancer xenografts derived from A549-IRR cells were resistant to IR treatment whereas xenografts derived from A549-P were sensitive to IR treatment (**Figure S7C**). BKA-073 repressed xenografts derived from either A549-P or A549-IRR cells, indicating that BKA-073 is also efficacious in radioresistant lung cancer xenografts.

### Combination of BKA-073 with Bcl2 inhibitor venetoclax (ABT-199) synergistically suppresses lung cancer in vitro and in vivo

**Figure 9:**
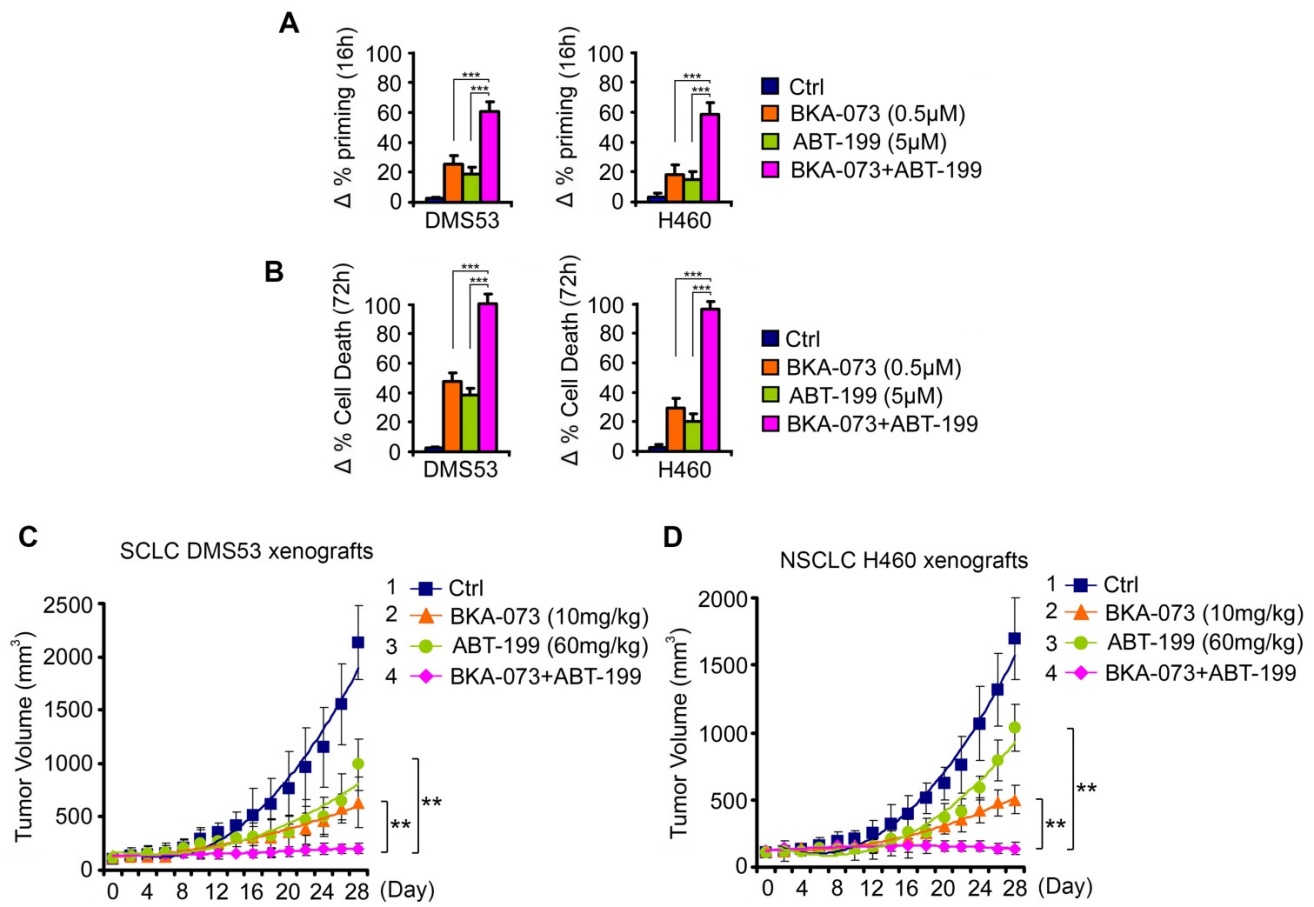
**BKA-073 synergizes with Bcl-2 inhibitor ABT-199 (venetoclax) against SCLC and NSCLC *in vitro* and *in vivo***. (**A-B**) SCLC DMS53 and NSCLC H460 cells were treated with BKA-073 (0.5μM), ABT-199 (5μM) or in combination for 16h or 72h, followed by analysis of dynamic BH3 profiling (A) or apoptotic cell death (B), respectively. Data represent the mean ± SD, *n* = 3 per group. ***P < 0.001, by 2-tailed *t* test. (**C-D**) Nu/Nu mice carrying SCLC DMS53 xenografts (C) or NSCLC H460 xenografts (D) were treated with BKA-073 (10mg/kg/d) i.p., ABT-199 (60mg/kg/d) orally, or the combination for 28 days. Tumor volume was measured once every 2 days. After treatment, mice were sacrificed and tumors were removed and analyzed. Data represent the mean ± SD, *n* = 6 per group. **P < 0.01, by 2-tailed *t* test.

To test whether direct activation of the proapoptotic activity of Bak combined with inhibition of the antiapoptotic function of Bcl2 achieves synergistic effects in lung cancer therapy, a SCLC cell line (DMS53) and NSCLC cell line (H460) that express endogenous Bcl2 and Bak were treated with venetoclax in combination with BKA-073 for 16h and 72h, followed by analysis of dynamic BH3 profiling and apoptosis, respectively. BKA-073 in combination with venetoclax exhibited strong synergism in the induction of mitochondrial priming and apoptosis in both SCLC and NSCLC lines (**Figure 9A, B**). To further test the synergy between BKA-073 and venetoclax *in vivo*, SCLC xenografts derived from DMS53 cells and NSCLC xenografts derived from H460 cells were treated with BKA-073 (10mg/kg/d) i.p., venetoclax (60mg/kg/d) orally, or the combination for 4 weeks. Results revealed that combined treatment with BKA-073 and venetoclax not only synergistically suppressed both SCLC and NSCLC *in vivo* (**Figure 9C, D**), but also was well tolerated without significant toxicity to mice (**Figure S8**).

### Higher levels of Bak in tumor tissues are correlated with poor prognosis of patients with NSCLC

**Figure 10:**
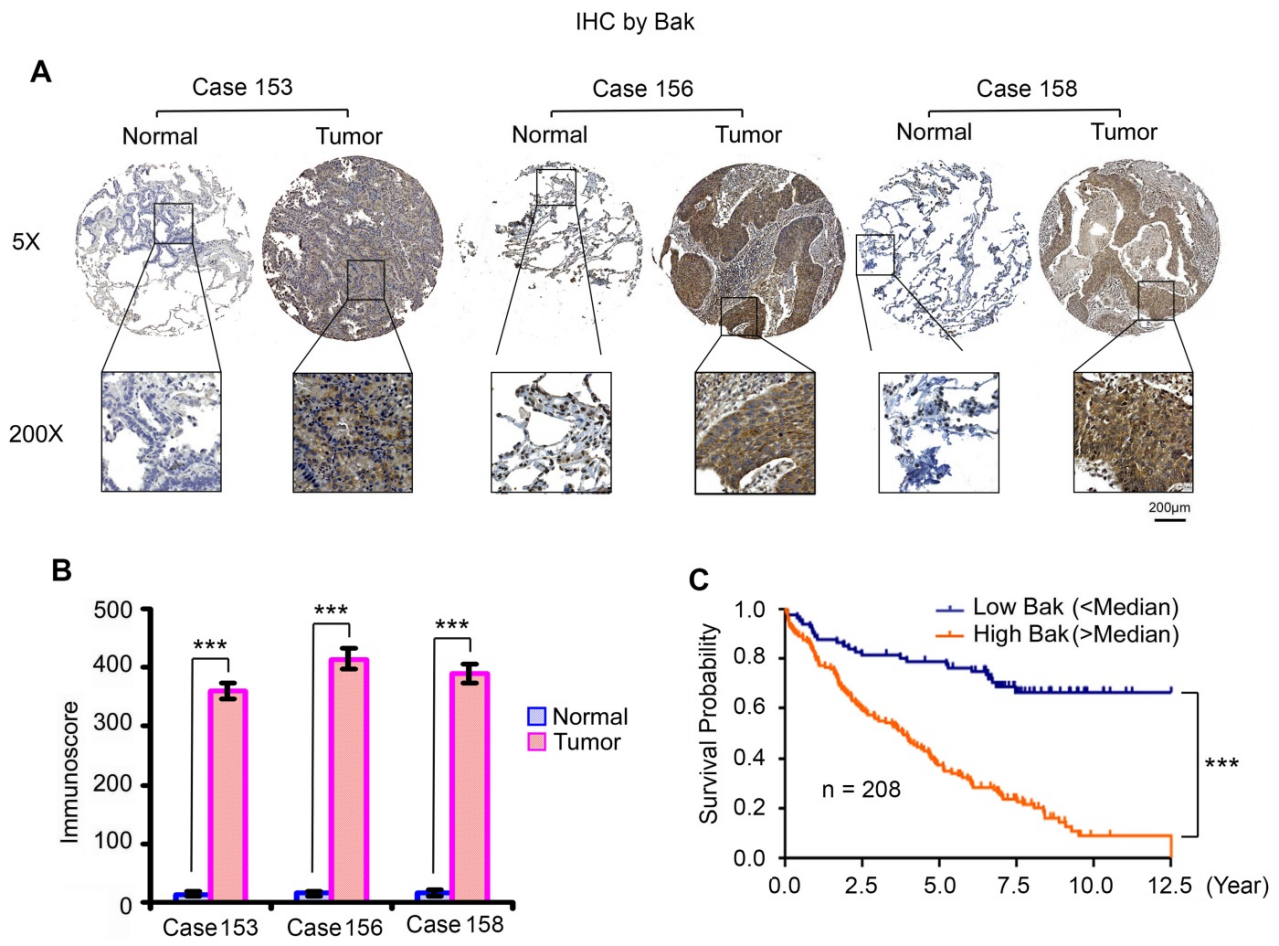
** High levels of Bak expression are associated with poor prognosis in NSCLC patients**. (**A**) Bak expression in normal lung tissues versus lung cancer tissues from three representative cases was analyzed by IHC using Bak antibody. Normal tissues are the adjacent normal lung tissues from the same cases. (**B**) Bak expression was quantified by immunoscore. Data represent the mean ± SD. ***P < 0.001, by 2-tailed *t* test. (**C**) Kaplan-Meier survival curve of NSCLC patients, n = 208. ***P < 0.001, by log-rank test.

Higher levels of endogenous Bak expression were observed in various human lung cancer cell lines, which did not cause apoptosis in cell culture medium without any treatment (**Figure 1**). This indicates that Bak protein is an inactive form under normal growth conditions. To further test whether Bak is upregulated in tumor tissues from NSCLC patients, we analyzed Bak expression in samples from 208 NSCLC patients by IHC staining using Bak antibody. We obtained formalin-fixed and paraffin-embedded human tissue samples from the tissue bank at Emory University Winship Cancer Institute. Tissue microarray (TMA) was constructed with replicate cores of tumor and adjacent normal lung. The semiquantitative evaluation of IHC staining of Bak was carried out using an immunoscore based on both the percentage of stained cells and staining intensity as described 35, 54, 55. Bak protein expression was significantly higher in tumor tissues compared to adjacent normal lung tissues (**Figure 10A, B**). Importantly, increased Bak expression in tumor tissues were correlated with poor prognosis of NSCLC patients (**Figure 10C**), suggesting that Bak is a potential prognostic biomarker for NSCLC. These data from patients suggest that Bak protein is an attractive target in lung cancer therapy. Development of the novel small molecule Bak activator (BKA-073) may represent an effective strategy to improve the outcome of NSCLC patients.

## Discussion

Bak is one of the major pro-apoptotic proteins required for programmed cell death (*i.e.* apoptosis) and apoptosis induction in cancer cells. Our findings have demonstrated that elevated Bak expression is correlated with poor prognosis in lung cancer, suggesting that Bak could be a promising prognostic indicator and a potential therapeutic target in lung cancer patients. Understanding the cellular mechanisms required for Bak activation is of great significance because of the potential to exploit this knowledge to develop new approaches and agents for cancer therapy. Here we identified BKA-073 as a small molecule Bak activator that targets the BH3 domain of Bak, activates the pro-apoptotic function of Bak, and exhibits potent antitumor activity against lung cancer. First, BKA-073 directly binds to Bak protein and induces Bak oligomerization in mitochondria leading to activation of Bak's proapoptotic function. Second, BKA-073-induced Bak oligomerization promotes mitochondrial priming and Cyt c release, which are early changes in net pro-apoptotic signaling at the mitochondria 24, 56. Finally, BKA-073-induced mitochondrial priming and Cyt c release leads to apoptotic cell death of lung cancer cells. Knockout of Bak but not Bax results in BKA-073 resistance in lung cancer cells and in lung cancer xenografts, indicating that the anti-tumor activity of BKA-073 occurs in a Bak-dependent manner. Furthermore, exogenous expression of WT but not the ∆BH3 mutant Bak in A549 Bak ^-/-^ cells can restore sensitivity to BKA-073, indicating that the apoptotic effect of BKA-073 requires its binding to the BH3 domain in the Bak protein. These findings suggest a mechanistic model of using a small-molecule Bak activator for cancer therapy.

Several Bax/Bak-independent mechanisms of apoptotic cell death have been previously described, including induction of ΔΨ_m_ breakdown and ROS production 57, serine protease(s)-dependent mechanism 58, caspase-8- or caspase-2-mediated mitochondria-independent downstream caspase activation 59, mitoptosis 60, etc. In our studies, a small percentage of apoptotic cell death (~20%) was still observed in the Bak^-/-^ and DKO A549 cells. It is possible that, in addition to the major Bak-dependent apoptosis mechanism, BKA-073 induces a small proportion of apoptotic cell death (~20%) through Bax/Bak-independent mechanism(s).

BKA-073 exhibited potent antitumor activity against lung cancer via induction of Bak activation (*i.e.* oligomerization) and apoptotic cell death in xenografts derived from either a lung cancer cell line or a patient-derived SCLC tumor. A dose range between 5 and 15 mg/kg/day was effective without weight loss or significant organ toxicities. Since BKA-073 suppressed the growth of PDX raised from two patients with refractory SCLC, this suggests that BKA-073 may have potential clinical utility for patients in the future.

Genetically engineered mouse models (GEMMs) develop *de novo* tumors in a natural immune proficient microenvironment. Tumors arising in advanced GEMMs closely mimic the histopathological and molecular features of their human counterparts. Thus, GEMMs are the most sophisticated animal models of human cancer, which closely recapitulate the pathophysiological process of human malignancies in genetically precisely defined systems 61. Bak is significantly upregulated in tumor tissues isolated from genetically engineered LSL-KRAS^G12D^ LKB1^fl/fl^ (KL) mice, further supporting BKA-073, a Bak agonist, as an ideal agent for treatment of KRAS-driven lung cancer. As expected, BKA-073 significantly reduced tumor burden in the lungs of KL mice leading to prolonged survival when compared with the untreated control group, suggesting that BKA-073 has the potential to improve the prognosis of patients with mutant KRAS-driven lung cancer.

Radiotherapy is a major therapeutic intervention for patients with lung cancer and is administered to up to 75% of patients with lung cancer during the course of their disease 62. A major challenge affecting the outcomes of patients with lung cancer is the development of acquired radioresistance. We observed increased levels of Bak protein in radioresistant lung cancer cell lines. Importantly, BKA-073 can overcome radioresistance effectively *in vitro* and *in vivo*, suggesting that BKA-073 may have the potential to be developed for the treatment of patients with radioresistant lung cancer.

Synergistic drug combinations can be rationalized using knowledge of single-drug action mechanisms. Venetoclax (ABT-199) is an FDA-approved selective Bcl-2 inhibitor for the treatment of CLL and AML 11, 12, 63, 64. However, venetoclax has limited potency in the treatment of lung cancer. Here we found that combined treatment with BKA-073 and venetoclax exhibited strong synergistic activity against both SCLC and NSCLC *in vitro* and *in vivo* without significant normal tissue toxicity. It is possible that inhibition of Bcl2 by venetoclax indirectly activates Bax to induce apoptosis, and the combination of BKA-073 and venetoclax activates both Bak and Bax leading to more apoptotic cell death compared to single agent alone. We propose that co-targeting Bcl2 and Bak may offer a more effective approach for lung cancer therapy.

In summary, we have discovered BKA-073 as a potent Bak activator that selectively targets the BH3 domain of Bak protein leading to Bak activation via its oligomerization, which promotes mitochondrial priming, Cyt c release, and apoptotic cell death. BKA-073 exhibits potent efficacy against both SCLC and NSCLC in xenografts, PDX, and GEMM models. BKA-073 not only overcomes radioresistance but also synergizes with Bcl-2 inhibitor venetoclax against lung cancer. Therefore, development of the Bak agonist as a class of anti-cancer agent potentially offers an effective strategy for lung cancer therapy.

## Abbreviations

BKA-073: Bak activator-073
DBP: dynamic BH3 profiling
Cyt c: cytochrome c
PDX: patient-derived xenograft
AdeCre: adenovirus Cre recombinase
GEMM: genetically engineered mouse model
i.p.: intraperitoneally
WBC: white cells
RBC: red blood cells
PLT: platelets
IHC: immunohistochemistry
SCLC: small cell lung cancer
NSCLC: non-small cell lung cancer
