# Supplementary material for: Discovery of Small Molecule Bak Activator for Lung Cancer Therapy
Source: Theranostics. 2021 Jul 25;11(17):8500–16. doi: 10.7150/thno.60349 (PMC8344021; doi:10.7150/thno.60349)
Supplement: Supplementary file 1 — Supplementary figures, materials and methods. [file thnov11p8500s1.pdf]

Supplementary Data and Supplementary Methods

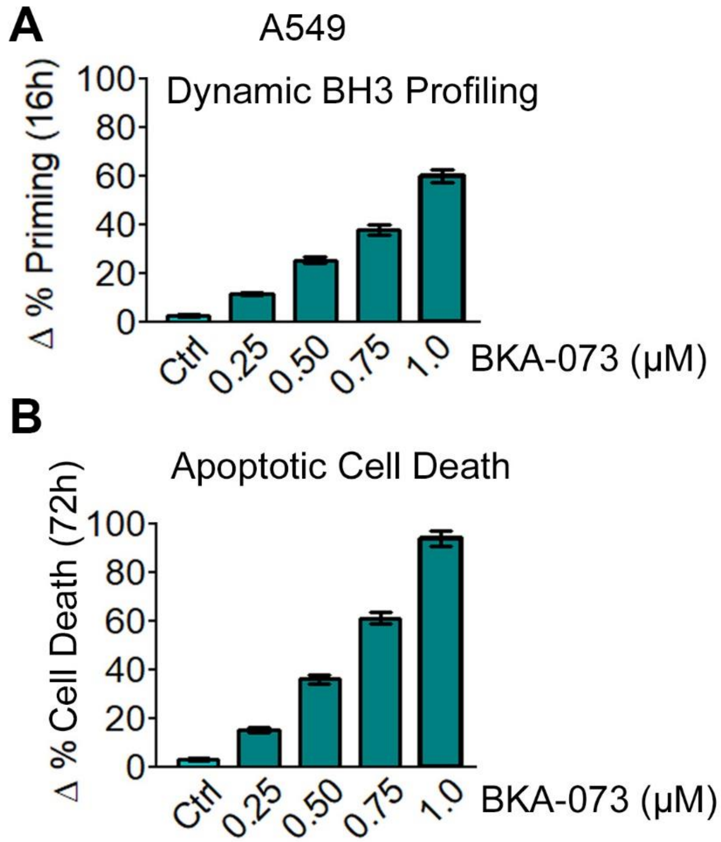

Figure S1. BKA-073 induces mitochondrial priming and apoptosis in a dose-dependent manner. (**A** and **B**) A549 cells were treated with increasing concentrations of BKA-073 (0, 0.25, 0.5, 0.75, 1μM) for 16h or 72h, followed by analysis of dynamic BH3 profiling (A) or apoptotic cell death (B), respectively. Data represent the mean ± SD, *n* = 3 per group.

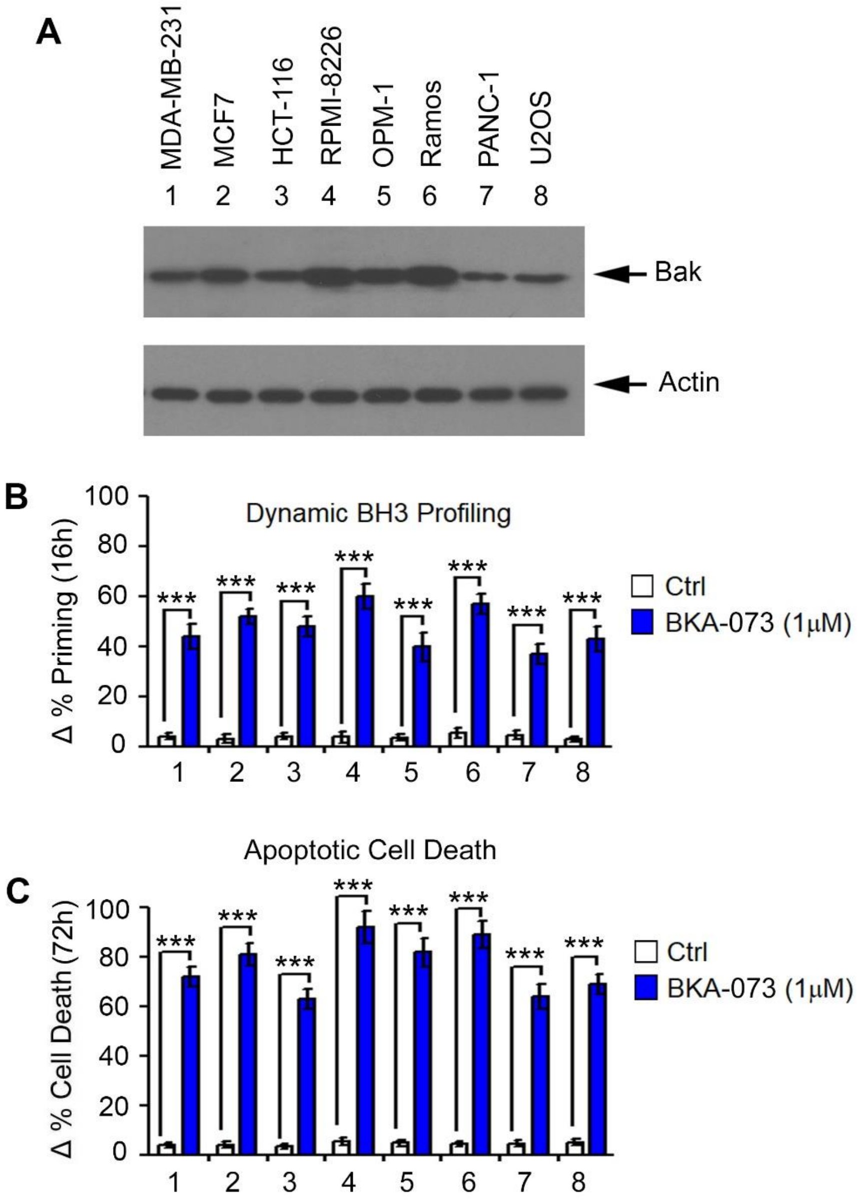

Figure S2. BKA-073 induces mitochondrial priming and apoptosis in various type of cancers. (**A**) Expression levels of Bak were analyzed by Western blot in various type of cancer cell lines. (**B-C**) Various type of cancer cell lines were treated with BKA-073 (1μM) for 16h or 72h, followed by analysis of dynamic BH3 profiling (B) or apoptotic cell death (C), respectively. Data represent the mean ± SD, *n* = 3 per group. ***$P < 0.001$, by 2-tailed *t* test.

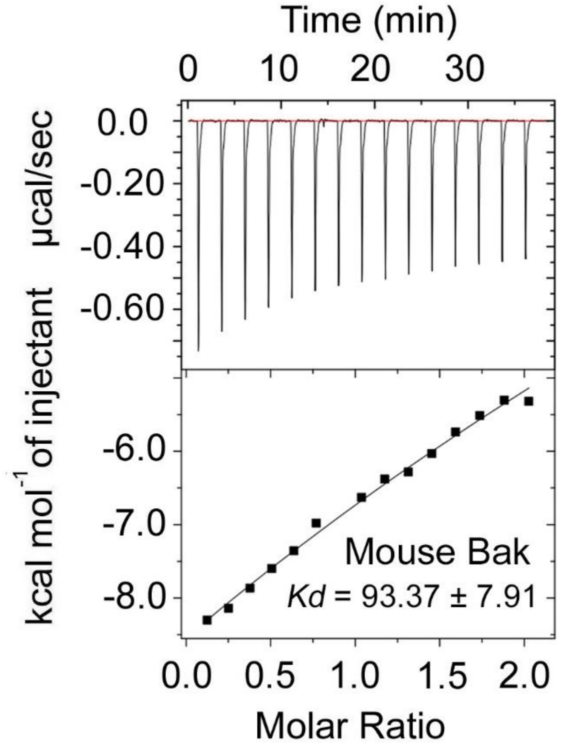

Figure S3. BKA-073 directly binds to mouse Bak protein in vitro. The binding affinity of BKA-073 with mouse wild type Bak protein was examined by isothermal titration calorimetry assay. The binding constant (K_D) value was determined by fitting of the titration curve to a 1-site binding mode.

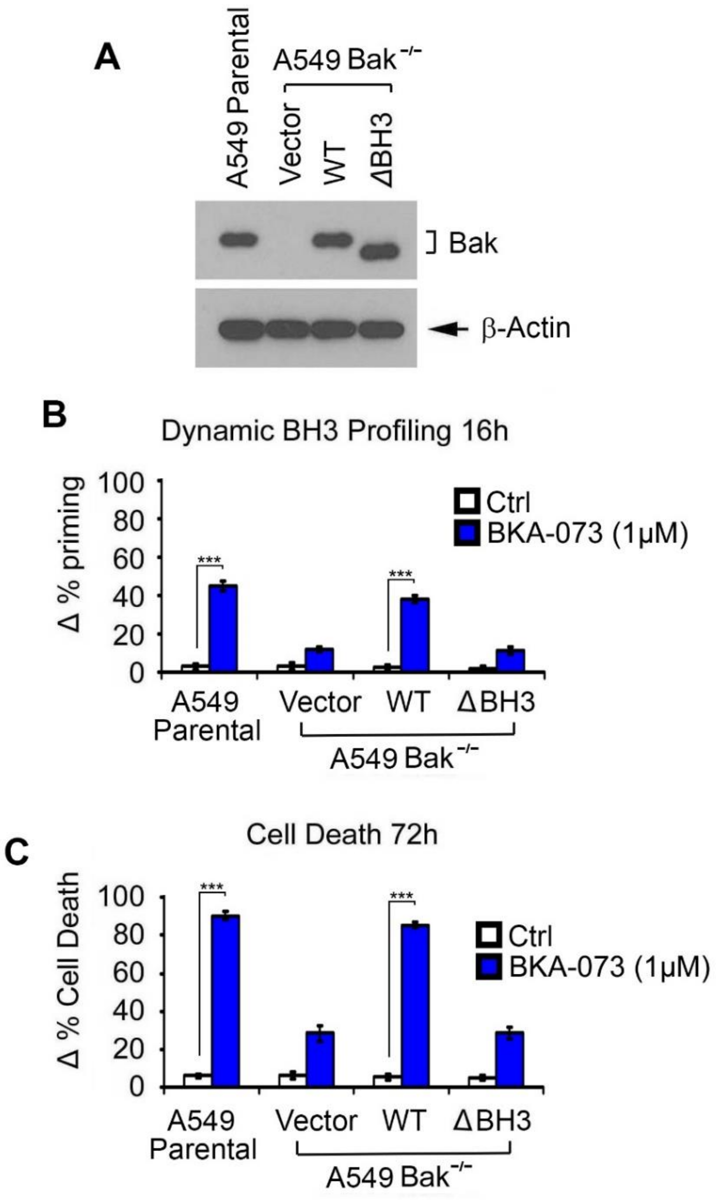

Figure S4. The BH3 domain of Bak is required for BKA-073 induction of mitochondrial priming and apoptotic cell death. (**A**) WT Bak, ΔBH3 mutant Bak or empty vector control were exogenously transfected into A549 Bak $^{-/-}$ cells, followed by Western blot using Bak antibody. (**B-C**) Parental A549 or A549 Bak $^{-/-}$ cells expressing exogenous WT Bak, ΔBH3 mutant Bak or empty vector control were treated with BKA-073 (1μM) for 16h or 72h, followed by analysis of dynamic BH3 profiling (B) or apoptotic cell death (C), respectively. Data represent the mean ± SD, n = 3 per group. ***P < 0.001, by 2-tailed t test.

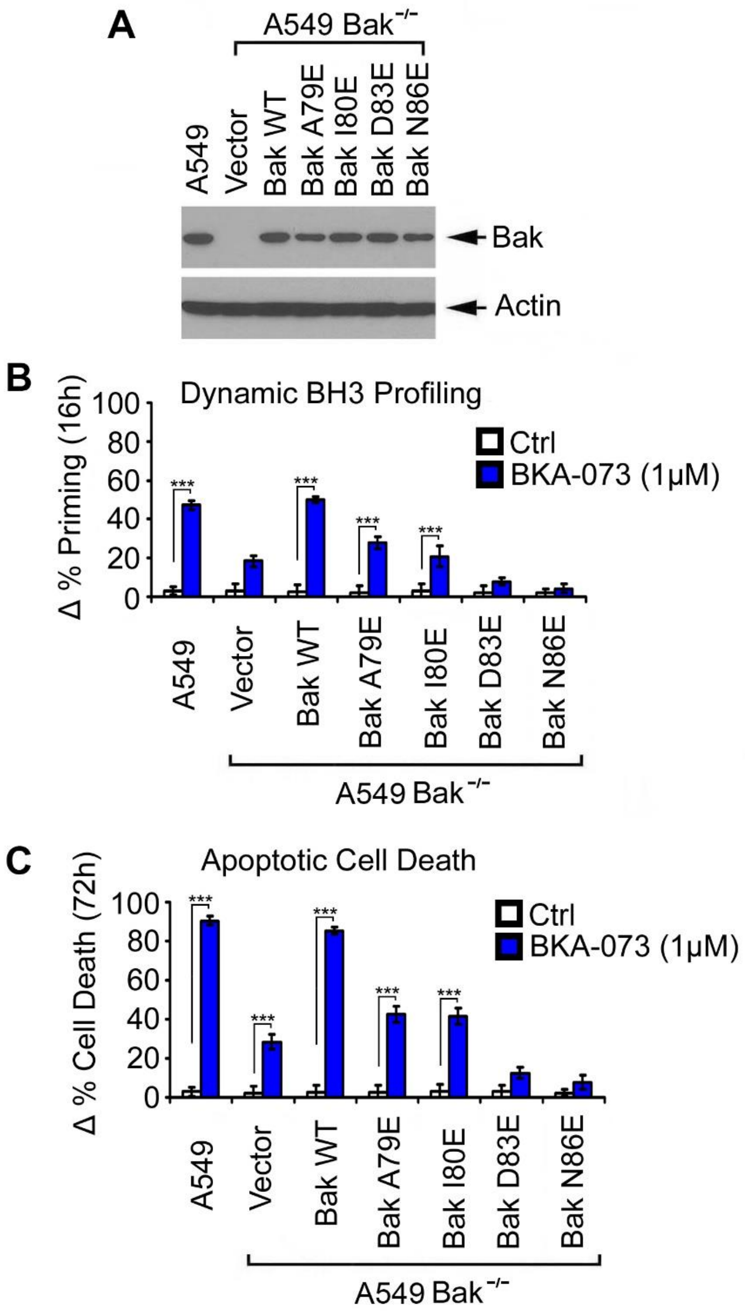

Figure S5. Binding to amino acids in the BH3 domain of Bak is essential for BKA-073 induction of mitochondrial priming and apoptotic cell death. (**A**) Bak WT, A79E, I80E, D83E and N86E Bak mutants or empty vector control were exogenously transfected into A549 Bak $^{-/-}$ cells, followed by Western blot using Bak antibody. (**B-C**) Parental A549 or A549 Bak $^{-/-}$ cells expressing exogenous Bak WT, Bak mutant(s) or empty vector control were treated with BKA-073 (1 μM) for 16h or 72h, followed by analysis of dynamic BH3 profiling (B) or apoptotic cell death (C), respectively. Data represent the mean ± SD, n = 3 per group. ***P < 0.001, by 2-tailed t test.

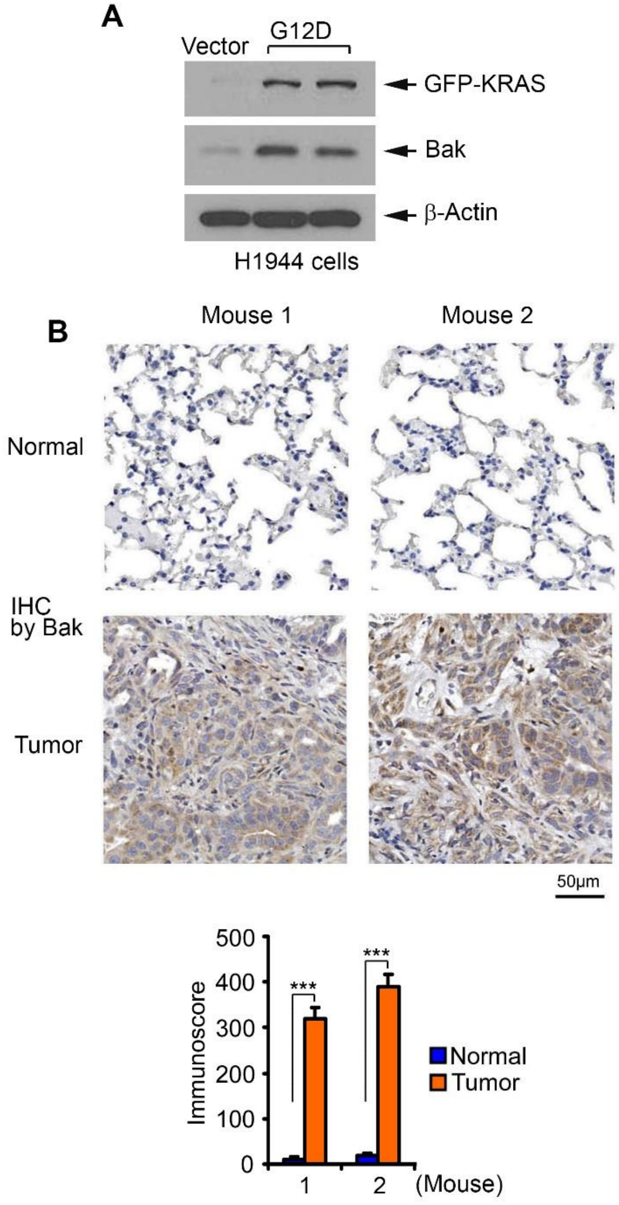

Figure S6. Bak expression is upregulated in mutant KRAS-driven lung cancer. (**A**) GFP-G12D KRAS was transfected into H1944 cells, followed by Western blot using GFP antibody. (**B**) IHC staining of Bak in tumor tissues versus adjacent normal lung tissue from two representative KL mice, which were quantified by analyzing immunoscore. Data represent the mean ± SD, n = 3 per group. ***P < 0.001, by 2-tailed t test.

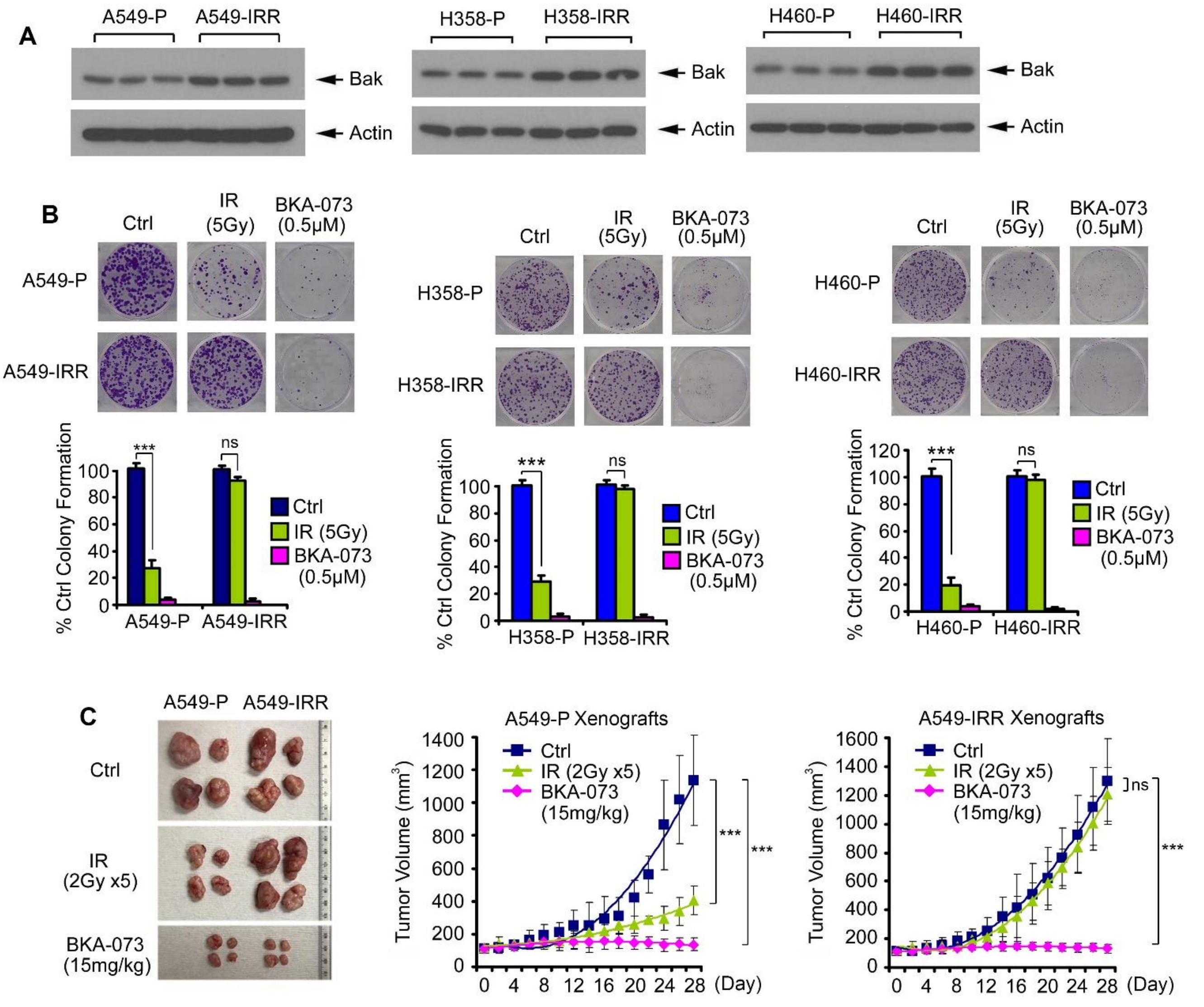

Figure S7. BKA-073 overcomes radioresistance of lung cancer *in vitro* and in vivo. (**A**) Expression levels of Bak in A549-P versus A549-IRR cells, H358-P versus H358-IRR cells or H460-P versus H460-IRR cells were analyzed by Western blot. (**B**) A549-P, A549-IRR, H358-P, H358-IRR, H460-P and H460-IRR cells were treated with IR (5Gy), BKA-073 (0.5 μM), followed by colony formation assay. Data represent the mean ± SD, n = 3 per group. ***P < 0.001, ns: not significant, by 2-tailed t test. (**C**) Nu/Nu mice carrying xenografts derived from A549-P or A549-IRR were treated with BKA-073 (15mg/kg/d) i.p. for 28 days or IR (2Gy × 5). Tumor volume was measured once every 2 days. After treatment, mice were sacrificed and tumors were removed and analyzed. Data represent the mean ± SD, n = 4 per group. ***P < 0.001, ns: not significant, by 2-tailed t test.

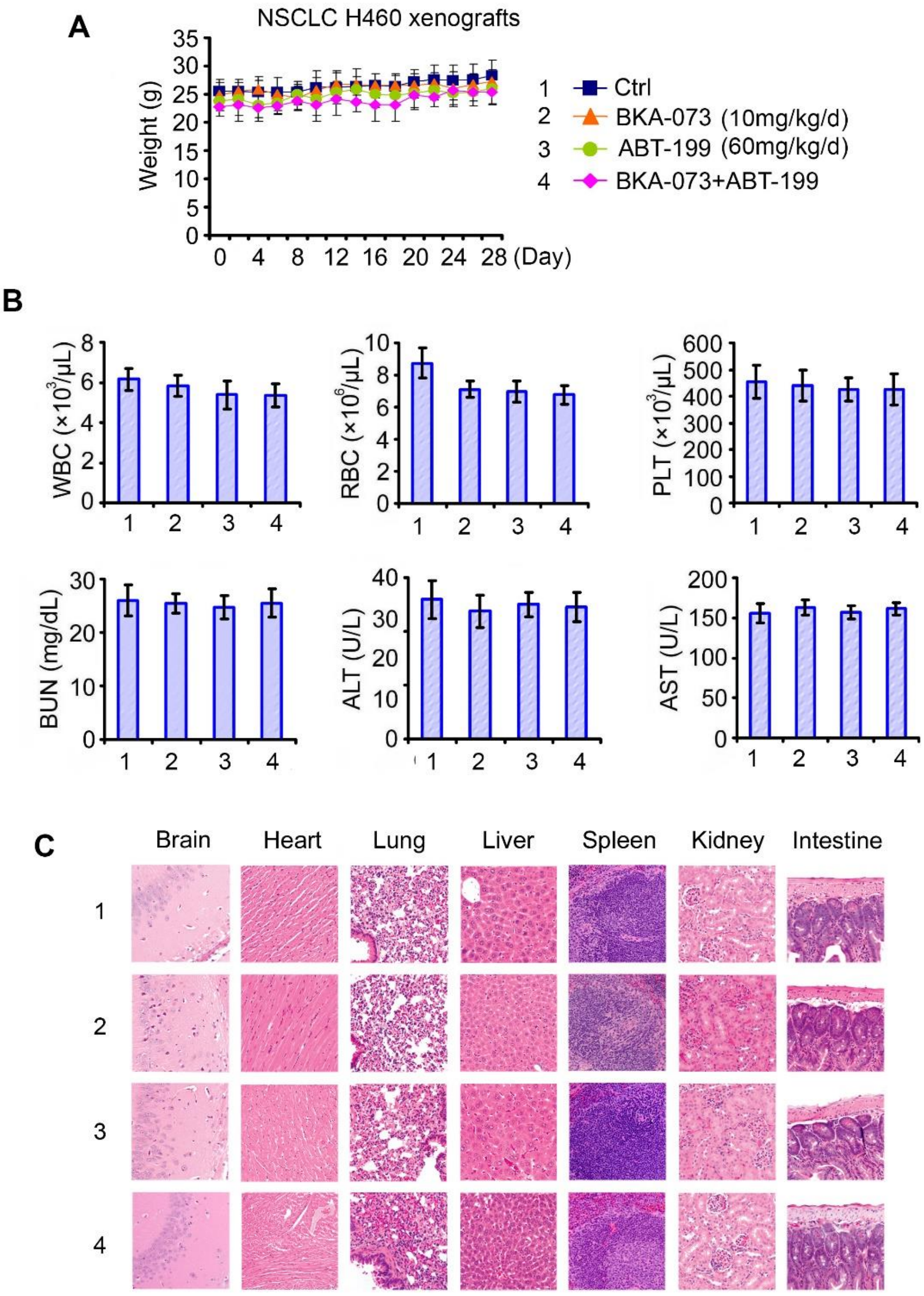

Figure S8. Toxicity of combined treatment with BKA-073 and ABT-199 *in vivo*. (**A**), (**B**) and (**C**) Body weight, blood analysis and H&E histology of various organs from mice carrying H460 xenografts after treatment with BKA-073 (10mg/kg/d), ABT-199 (60mg/kg/d) or in combination for 28 days.

Supplemental Materials and Methods

Materials

Small molecule NSC14073 (BKA-073) was obtained from the Drug Synthesis and Chemistry Branch, Developmental Therapeutic Program, Division of Cancer Treatment and Diagnosis, National Cancer Institute (NCI, Bethesda, MD) (http://dtp.nci.nih.gov/RequestCompounds). Venetoclax (ABT-199, 205807) was purchased from MedKoo Biosciences (Morrisville, NC). Antibodies including PARP (sc-8007), Bax (sc-7480), Mcl-1 (sc-12756), cytochrome c (sc-13156) and actin (sc-47778) were purchased from Santa Cruz (Santa Cruz, CA). Bcl2 antibody (05-826) was obtained from Calbiochem (Darmstadt, Germany). Bcl-XL (ab32370), Bak (ab32371) and Bax (ab104156) antibodies were purchased from Abcam (Cambridge, MA). Active caspase 3 (9579S) and Mcl-1 (94296S) antibodies were purchased from Cell Signaling Technology (Danvers, MA). Fluorescent Bak BH3 domain peptide (FAM-GQVGRQLAIIGDDINR) and purified Bcl-XL protein were purchased from NeoBioSci™ (Cambridge, MA). Bim BH3 peptide (AS-62278) was obtained from AnaSpec, Inc. (Fremont, CA). Purified recombinant human Bak protein (BAK1-26166TH) was purchased from Creative Biomart (Shirley, NY). Purified recombinant human Bax protein (H00000581-P01) was obtained from Novus Biologicals (Littleton, CO) and purified human Bcl-XL protein (SRP0187) was purchased from Sigma-Aldrich (St. Louis, MO). Purified human Bcl2 protein was obtained from Protein*X* Lab (San Diego, CA). Purified recombinant human Mcl-1 protein (GWB-P0342D) was purchased from GenWay Biotech, Inc. (San Diego, CA). Purified recombinant human Bcl-w (824-BW-050) protein was obtained from R&D systems (Minneapolis, MN). Bis (maleimido) hexane (BMH) (22330) was purchased from Thermo Scientific (Rockford, IL). JC-1 (ENZ-52304) was obtained from Enzo Life Sciences, Inc. (Farmingdale, NY). All other reagents used were obtained from commercial sources unless otherwise stated.

Preparation of cell lysate and Western blot

Cells were washed with cold PBS and resuspended in ice-cold EBC buffer (0.5% Nonidet P-40, 50mM Tris, pH 7.6, 120mM NaCl, 1mM EDTA, and 1mM-β-mercaptoethanol) containing protease inhibitor mixture set I. Following cell lysis by sonication and centrifugation at 14,000 x g for 15 min at 4 °C, the resulting supernatant was collected as the total cell lysate. Western blot was performed by loading 50μg of protein per lane on an 8-12% SDS-PAGE, followed by protein transfer to nitrocellulose membrane for analysis of specific protein(s) as previously described [1].

Sulforhodamine B (SRB) colorimetric assay

Cells were seeded at a density of 5×10^3 per well in 96-well plates and allowed to grow overnight. After treatment with BKA-073, the surviving cell fraction was determined using the sulforhodamine B (SRB) assay as described [2, 3]. Optical density (OD) was measured at 510nm in a microplate reader (Synergy H1, BioTek®, Winooski, VT).

Apoptotic cell death assay

Apoptotic cells were detected using Annexin-V Apoptosis Detection Kit (556547, BD Biosciences, San Jose, CA) according to the manufacturer's instructions. After treatment with BKA-073 or other agent(s) for 72h, cells were washed twice with cold 1x PBS and then resuspended in 1 x Binding Buffer at a concentration of 1×10^6 cells/ml. 100 μl of the solution

(1 x 10^5 cells) was transferred to a 5 ml culture tube, followed by the addition of 5 µl of FITC Annexin V and 5 µl propidium iodide (PI). The cells were gently vortexed and incubated for 15 min at RT (25°C) in the dark. 400 µl of 1x binding buffer was added to each tube. Samples were analyzed by flow cytometry (BD FACSCanto™II, BD Biosciences, San Jose, CA). Δ% cell death was calculated with the formula: (% cell death ^{treated} - % cell death $^{non-treated}$) as described [4-6].

Fluorescence polarization (FP) assay
Fluorescence polarization assay was carried out as described previously [7]. Briefly, 3 nM of fluorescent Bak BH3 domain peptide was incubated with Bak protein (6 nM) or other Bcl-2 family member(s) in the absence or presence of increasing concentrations of BKA-073 in the binding buffer [50 mM Tris (pH 8.0), 150 mM NaCl, 0.1% bovine serum albumin (BSA), and 5 mM DTT] in black 96-well microplates. The plate was mixed on a shaker for 1 minute and incubated at room temperature for an additional 15 minutes. The FP values (in millipolarization units) were measured using a fluorescence microplate reader at 485/530 nm (Gemini XPS, Molecular Devices). Data analysis and inhibitory constant (Ki) value was determined by GraphPad prism software as described [7].

Cloning, protein expression and purification of mouse Bak protein
The clone pMIH-mRuby2-mouse Bak (#111627) for generation of mouse Bak protein was obtained from Addgene (Watertown, MA). The mouse Bak cDNA was cloned into pET29b expression vector between BglII and XhoI sites as His tag fusions. The BL21 DE3 Escherichia coli containing plasmid was grown at 37°C in LB (Luria-Bertani) media. The protein expression was induced with IPTG (0.2 mM) at 18°C for 16 h when the density reached an OD600 of 0.6. Bacteria were then harvested and resuspended in 30 ml buffer A (20 mM Tris, pH 8.0, 120 mM NaCl, 10% glycerol, and protease inhibitor cocktail) and lysed by sonication. The lysate was centrifuged at 14,000 rpm for 30 min. The resulting supernatant was incubated with HisPur Cobalt Resin (Thermo Scientific) at 4 °C for 2h. After washing with buffer A, His-tagged mouse Bak proteins was eluted with buffer B (20 mM Tris, pH 8.0, 120 mM NaCl, 300 mM imidazole, 10% glycerol with protease inhibitor). The elution peak of interest was checked by SDS-PAGE electrophoresis. The purified mouse Bak protein was dialyzed with buffer A. This purified mouse Bak protein was used for analysis of mouse Bak/BKA-073 binding with isothermal titration calorimetry (ITC) assay.

Subcellular fractionation
Cells (2 $\times 10^7$) were washed with cold 1× PBS and resuspended in isotonic mitochondrial buffer (210 mM mannitol, 70 mM sucrose, 1 mM EGTA, 10 mM Hepes, pH 7.5) containing 10% protease inhibitor mixture set I, homogenized with a polytron homogenizer, and then centrifuged at 1000 × *g* for 5 min to remove the nuclei and unbroken cells. The supernatant was centrifuged at 13,000 × *g* for 10 min to pellet mitochondria. The resulting supernatant is the cytosolic fraction. Mitochondria were washed twice with mitochondrial buffer and resuspended in 1% Nonidet P-40 lysis buffer, rocked for 60 min, and then centrifuged at 17,530 × *g* for 10 min at 4 °C. The resulting supernatant containing mitochondrial proteins was collected. Protein (50 µg) from each fraction was subjected to SDS-PAGE. Cyt c was analyzed by Western blot.

Knockout of Bak or Bax by CRISPR/Cas9

Human Bak CRISPR/Cas9 knockout plasmid (sc-400646) and human Bax CRISPR/Cas9 knockout (sc-400042) plasmid were purchased from Santa Cruz (Santa Cruz, CA). Human Bak CRISPR/Cas9 knockout plasmid is a pool of 3 different gRNA plasmids, including sc-400646 A, 5’-GCA TGA AGT CGA CCA CGA AG-3’; sc-400646 B, 5’-GTT GAT GTC GTC CCC GAT GA-3’; sc-400646 C, 5’-GCT CAC CTG CTA GGT TGC AG-3’. Human Bax CRISPR/Cas9 KO plasmid is a pool of 3 different gRNA plasmids, including sc-400042 A, 5’-TTT CTG ACG GCA ACT TCA AC -3’; sc-400042 B, 5’- CCA TTC GCC CTG CTC GAT CC-3’; sc-400042 C, 5’-AGC GAG TGT CTC AAG CGC AT-3’. A549 cells were transfected with human Bak CRISPR/Cas9 KO plasmids or Bax CRISPR/Cas9 KO plasmids using Nanojuice (71902, EMD Millipore). After 48h, the GFP positive cells were sorted and plated in 96-well plates at one cell per well by flow cytometry. The successful knockout of Bak or Bax was confirmed by Western blot using Bak or Bax antibody, respectively.

Mutagenesis and cloning of Bak

The WT-Bak cDNA in pMXs-IG was kindly provided by Dr. Ruth Kluck (Walter and Eliza Hall Institute of Medical Research) and was subcloned into lentivirus vector pCDH-CMV-Puro (CD510B-1, System Biosciences, Palo Alto, CA) between the EcoRI and Not I sites. The BH3 deletion Bak mutant (ΔBH3) and a panel of Bak mutants within the BH3 domain at the specific residues that were identified by the initial docking simulations, including A79E, I80E, D83E and N86E Bak mutants, were generated by inverse PCR using the WT Bak in pCDH-CMV-Puro as template. Primers used for PCR were as follows: ΔBH3: forward, 5’-TCA GAG TTC CAG ACC ATG TTG CAG C-3’, reverse, 5’- CTG CCC CAT GGT GCT GCT AGG TTG C-3’; A79E: forward, 5’-GGG CAG GTG GGA CGG CAG CTC GAA ATC ATC GGG GAC GAC ATC AAC C-3’, reverse, 5’-G GTT GAT GTC GTC CCC GAT GAT TTC GAG CTG CCG TCC CAC CTG CCC; I80E: forward, 5’- CAG GTG GGA CGG CAG CTC GCC GAA ATC GGG GAC GAC ATC AAC CGA-3’, reverse, 5’- TCG GTT GAT GTC GTC CCC GAT TTC GGC GAG CTG CCG TCC CAC CTG; D83E: forward, 5’- CGG CAG CTC GCC ATC ATC GGG GAA GAC ATC AAC CGA CGC TAT GAC-3’, reverse, 5’- GTC ATA GCG TCG GTT GAT GTC TTC CCC GAT GAT GGC GAG CTG CCG-3’; N86E: forward, 5’- GCC ATC ATC GGG GAC GAC ATC GAA CGA CGC TAT GAC TCA GAG TTC-3’, reverse, 5’- GAA CTC TGA GTC ATA GCG TCG TTC GAT GTC GTC CCC GAT GAT GGC-3’. After PCR, the amplification products were digested with DpnI to remove the non-mutated WT template, subjected to 1% agarose gel and purified by Qiagen gel extraction kit. Purified PCR products were circularized by ligation using T4 DNA ligase, followed by transformation into DH5α for amplification. The mutants were confirmed by sequencing.

Generation of recombinant His-tagged Bak proteins

Human Bak WT and BH3 deletion mutant (ΔBH3) were cloned into the pET29b expression vector as His_6-tag fusions as previously described [8]. BL21 DE3 *Escherichia coli* containing each plasmid was grown at 37°C in LB (Luria-Bertani) media. When the density reached an OD600 of 0.6, protein expression was induced with IPTG (0.2 mM) at 18°C for 16 h. Bacteria were then harvested and resuspended in 30 ml buffer A (20 mM Tris, pH 8.0, 120 mM NaCl, 10% glycerol and protease inhibitor cocktail) per 1 L of bacteria and lysed by sonication. The lysate was centrifuged at 14,000 rpm for 30 min, and the supernatant was incubated with HisPur Cobalt Resin (Thermo Scientific) at 4 °C for 2h. After washing with buffer A, His-tagged

WT and ΔBH3 mutant Bak proteins were eluted with buffer B (20 mM Tris, pH 8.0, 120 mM NaCl, 300 mM imidazole, 10% glycerol with protease inhibitor). The elution peak of interest was checked by SDS-PAGE electrophoresis. The purified protein was dialyzed with buffer A for *in vitro* BKA-073 binding assay.

Transfection of WT and mutant Bak into cells

The pCDH-CMV-Puro plasmids carrying WT or mutant Bak were co-transfected into 293FT cells with a lentivirus packaging plasmid mixture (pCMV-dR8.2 dvpr and pCMV-VSV-G) (System Biosciences, CA) using the NanoJuice transfection kit (EMD Chemical, Inc.). After 72h, the virus-containing medium supernatant was harvested by centrifugation at 20,000 × g. A549 $Bak^{-/-}$ cells were infected with the virus-containing media in the presence of polybrene (8 μg/ml) for 24h. Stable positive clones were selected using 3 μg/ml puromycin. The expression of WT or mutant Bak was confirmed by western blot using a Bak antibody.

Immunohistochemical (IHC) staining

After deparaffinization, rehydration, inactivation of endogenous peroxidase, and antigen retrieval, IHC staining was performed using R.T.U. Vectastain Kit (Vector Laboratories) according to the manufacturer's instructions. Bak antibody (1:200) or active caspase 3 antibody (1:200) were used for IHC staining. Active caspase 3 positive cells in tumor tissues were scored at 400 × magnification. The average number of positive cells per 0.0625 mm^2 area was determined from three separate fields in each of three independent tumor samples as described [9]. The semiquantitative evaluation of IHC staining of Bak was carried out using an immunoscore based on both the percentage of stained cells and staining intensity as described [2]. The intensity was defined as follows: 0, no appreciable staining; 1, weak intensity; 2, moderate intensity; 3, strong intensity; 4, very strong intensity. The immunoscore was calculated by multiplying the intensity by the percentage of positive staining, producing a total range of 0 to 500.

Mouse blood analysis

Whole blood (250µL) was collected in EDTA-coated tubes via cardiac puncture of anesthetized mice for hematology studies. Specimens were analyzed for white blood cells (WBC), red blood cells (RBC), platelets (PLT), alanine aminotransferase (ALT), aspartate aminotransferase (AST) and blood urea nitrogen (BUN) in the Clinical Pathology Laboratory at the University of Georgia (Athens, GA).

References:

1. Han B, Park D, Li R, Xie M, Owonikoko TK, Zhang G, et al. Small-Molecule Bcl2 BH4 Antagonist for Lung Cancer Therapy. Cancer Cell. 2015; 27: 852-63.
2. Liu Y, Sun SY, Owonikoko TK, Sica GL, Curran WJ, Khuri FR, et al. Rapamycin induces Bad phosphorylation in association with its resistance to human lung cancer cells. Mol Cancer Ther. 2012; 11: 45-56.
3. Vichai V, Kirtikara K. Sulforhodamine B colorimetric assay for cytotoxicity screening. Nat Protoc. 2006; 1: 1112-6.

4. Ryan J, Letai A. BH3 profiling in whole cells by fluorimeter or FACS. Methods. 2013; 61: 156-64.
5. Montero J, Sarosiek KA, DeAngelo JD, Maertens O, Ryan J, Ercan D, et al. Drug-induced death signaling strategy rapidly predicts cancer response to chemotherapy. Cell. 2015; 160: 977-89.
6. Fraser C, Ryan J, Sarosiek K. BH3 Profiling: A Functional Assay to Measure Apoptotic Priming and Dependencies. Methods Mol Biol. 2019; 1877: 61-76.
7. Park D, Magis AT, Li R, Owonikoko TK, Sica GL, Sun SY, et al. Novel small-molecule inhibitors of Bcl-XL to treat lung cancer. Cancer Res. 2013; 73: 5485-96.
8. Moldoveanu T, Liu Q, Tocilj A, Watson M, Shore G, Gehring K. The X-ray structure of a BAK homodimer reveals an inhibitory zinc binding site. Mol Cell. 2006; 24: 677-88.
9. Oltersdorf T, Elmore SW, Shoemaker AR, Armstrong RC, Augeri DJ, Belli BA, et al. An inhibitor of Bcl-2 family proteins induces regression of solid tumours. Nature. 2005; 435: 677-81.
